# Gelatine-based foams produced by enzymatic foaming: formulation–structure relationships affecting expansion and stability

**DOI:** 10.1039/d6ra00030d

**Published:** 2026-03-02

**Authors:** Shwan Abdullah Hamad

**Affiliations:** a Pharmacy Department, College of Medicine, Komar University of Science and Technology Chaq-Chaq, Qularaisi Sulaymaniyah Kurdistan Region of Iraq shwan.abdulah@komar.edu.iq

## Abstract

Gelatine-based foams are attractive wound-dressing materials due to their biocompatibility, conformability, and exudate-absorptive capacity; however, most reported systems rely on mechanical or chemical foaming routes that offer limited control over expansion and structural stability under physiological conditions. Here, a biodegradable gelatine-based solidified foam is developed *via* catalase-mediated enzymatic oxygen generation followed by glutaraldehyde-induced network stabilisation, enabling rapid *in situ* foam formation and fixation at 37 °C. A systematic parametric study was conducted to elucidate the effect of gelatine, hydrogen peroxide, catalase, and glutaraldehyde concentrations on foam expansion and 24 h volume retention. Statistical analysis (one-way ANOVA with Tukey's post hoc test) showed that foam behaviour reflects the interplay among enzymatic gas generation, interfacial stabilisation by gelatine, and matrix stiffening through covalent cross-linking. Excessive gas generation or cross-link density reduced structural integrity, whereas intermediate formulation ranges produced foams with improved expansion–stability balance. A practical formulation window was identified (2.0–3.0 wt% gelatine, 3.0–4.0 wt% hydrogen peroxide, 0.2 wt% catalase, and 2.5–3.0 wt% glutaraldehyde), providing a favourable compromise between high expansion and sustained volume retention over 24 h. These results provide formulation-level design guidance for enzymatically generated biomedical foams and support their potential as flexible, absorbent wound-dressing materials.

## Abstract 2

Catalase-mediated oxygen generation enables rapid in situ formation of structurally stabilised gelatine foams with tunable expansion, providing a controllable enzymatic strategy for advanced wound-dressing materials.

## Graphical abstract

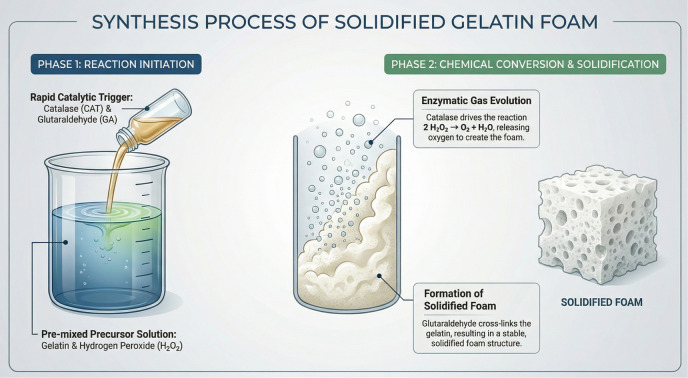

## 1 Introduction

Effective wound healing requires an optimal local environment that supports tissue regeneration, regulates hydration, manages exudate, and minimises infection risk. Acute wound repair proceeds through coordinated phases of inflammation, proliferation, and remodelling; however, this process is frequently disrupted by infection, malnutrition, impaired oxygenation, malignancy, nicotine exposure, or corticosteroid therapy, leading to chronic, non-healing wounds and increased morbidity.^1–3^ Maintenance of a moist wound environment is a critical determinant of healing efficiency, as demonstrated by Winter.^4^ At the same time, adequate oxygen availability remains essential for tissue repair despite infection, as microbial metabolism depletes oxygen and generates reactive species that delay healing.^5^

Modern wound dressings are therefore designed to balance hydration, absorption, and structural integrity while protecting against infection. Polyurethane film dressings with acrylic adhesives provide vapour permeability and periwound protection but often fail to adequately conform to irregular wound geometries or fill wound cavities,^6–8^ limiting their effectiveness in chronic wounds.^9–11,15^ Hydrocolloid and foam dressings offer improved exudate management; however, conventional foams frequently exhibit limited conformability and incomplete wound-bed contact,^13^ reducing therapeutic performance.^14,15^

Porous and gas-expanded polymeric systems have consequently attracted increasing interest due to their enhanced absorption capacity and ability to adapt to wound contours. Gas-evolving approaches, including carbon-dioxide-based foaming, have been used to generate superabsorbent hydrogels and macroporous polymer matrices with improved swelling and fluid uptake.^12,16,17^ Nevertheless, many reported systems rely on mechanical foaming, freeze-drying, or chemical gas-forming agents, offering limited control over pore formation and poor responsiveness to dynamic wound environments.^19^

Natural biopolymers, including gelatine, alginate, chitosan, and collagen, are particularly attractive for wound-care applications due to their biocompatibility, biodegradability, and intrinsic biological activity.^20–28^ Gelatine, in particular, combines haemostatic properties, surface activity, and the ability to form physically or chemically cross-linked networks, making it well suited for foam and hydrogel dressings.^35–38^ Incorporation of antimicrobial components, notably silver-based systems, further enhances dressing performance by reducing bioburden, an increasingly important requirement given the rise of antibiotic-resistant pathogens.^18,30–34^

Enzymatic gas generation offers an alternative strategy for foam formation under mild, physiologically relevant conditions. Catalase-mediated decomposition of hydrogen peroxide provides rapid and controllable oxygen evolution, enabling *in situ* foam expansion within a polymeric scaffold. When coupled with covalent cross-linking, enzymatic foaming can generate flexible, porous structures that are stabilised immediately after formation. In porous polymeric foams, macroscopic performance arises from the balance between gas-generation rate, interfacial stabilisation, and matrix solidification. However, systematic studies linking enzymatic gas generation with formulation parameters and post-formation foam stability under physiologically relevant conditions remain limited.

Here, we report a biodegradable, biocompatible gelatine-based foam fabricated *via* catalase-mediated enzymatic foaming and covalent stabilisation. A systematic formulation study was performed to quantify the individual and combined effects of gelatine, hydrogen peroxide, catalase, and glutaraldehyde concentrations on foam expansion and 24 h volume retention at physiological temperature, providing formulation-level design guidance for the development of flexible, absorbent wound-dressing materials.

## 2 Materials and methods

### 2.1 Materials

Porcine skin-derived gelatine (Sigma-Aldrich) was used as the biopolymer scaffold and foam stabiliser. Hydrogen peroxide solution (12% w/v, 40 volumes; Fisher Chemical) served as the gas-generating substrate, while catalase from bovine liver (Sigma-Aldrich) catalysed hydrogen peroxide decomposition. Glutaraldehyde solution (50 wt% in water; Sigma-Aldrich) was used as the covalent cross-linking agent. Ultrapure water was obtained using an Elgastat Prima reverse osmosis unit followed by a Milli-Q purification system and used for all formulations. All reagents were used as received.

### 2.2 Enzymatic foam preparation

Gelatine-based solidified foams were prepared *via in situ* enzymatic gas generation followed by covalent stabilisation. All experiments were conducted at 37 °C in a thermostatically controlled water bath. Each formulation was prepared in triplicate (*n* = 3).

Unless otherwise stated, a total formulation volume of 10 mL was used in a 100 mL glass beaker, corresponding to an initial solution height (*L*_soln_) of 0.55 cm. Foam generation experiments were initially performed in an open glass beaker to allow unrestricted oxygen release. In selected experiments, screw-cap vials were used as reaction vessels; however, the caps were left loose during foam formation and only secured after apparent setting (≈1–2 min). This ensured that gas evolution occurred under effectively open conditions.

Gelatine was dissolved in 8 mL of hydrogen peroxide solution at the desired concentration under gentle heating and stirring. A separate 2 mL solution containing catalase and glutaraldehyde at predefined concentrations was then rapidly added with manual mixing to initiate foam formation.

**Fig. 1:**
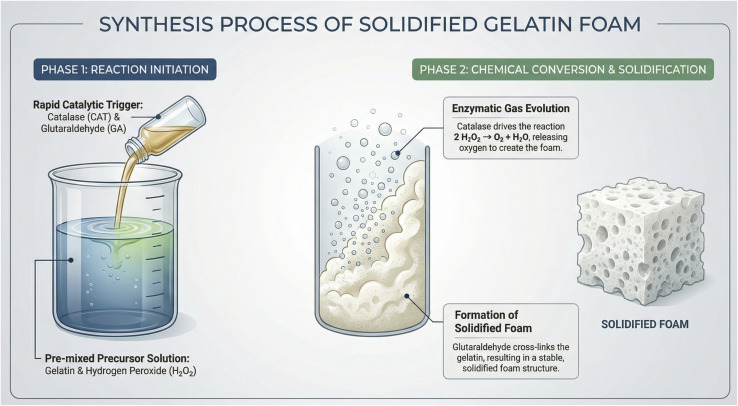
Schematic representation of the solidified gelatine foam preparation.

Catalase-mediated decomposition of hydrogen peroxide generated oxygen gas, inducing rapid volumetric expansion of the gelatine matrix, while simultaneous glutaraldehyde cross-linking stabilised the expanded structure at the macroscopic level. Foam expansion and solidification occurred within seconds under all conditions. For this study, “solidified foam” refers to a self-supporting structure that retained its macroscopic shape without observable flow under gravity at 37 °C. The overall gas-generation reaction involves the stoichiometric decomposition of 2 moles of hydrogen peroxide, yielding 1 mole of oxygen gas and 2 moles of water. A schematic illustration of the foam-formation mechanism is shown in Fig. 1.

### 2.3 Parametric optimisation of foam formulations

A sequential parametric optimisation strategy was employed, in which one formulation variable was varied at a time while all others were held constant. Balanced conditions identified in each study were carried forward to subsequent optimisation steps.

#### 2.3.1 Gelatine concentration

Gelatine concentrations of (1.0, 2.0, 3.0, 4.0, and 5.0) wt% were evaluated by dissolving gelatine in 8 mL of 3.0 wt% hydrogen peroxide, followed by the addition of 2 mL containing 0.2 wt% catalase and 2.5 wt% glutaraldehyde.

#### 2.3.2 Hydrogen peroxide concentration

Hydrogen peroxide concentrations of (1.2, 2.4, 3.6, 4.8, and 6.0) wt% were investigated using 5.0 wt% gelatine, 0.2 wt% catalase, and 2.5 wt% glutaraldehyde. A gelatine concentration of 5.0 wt% was selected as the baseline for subsequent parametric studies because it produced mechanically robust foams with improved handling reproducibility compared with lower concentrations.

#### 2.3.3 Catalase concentration

Catalase concentrations of (0.05, 0.10, 0.20, 0.30, and 0.40) wt% were examined using 5.0 wt% gelatine, 3.6 wt% hydrogen peroxide, and 2.5 wt% glutaraldehyde.

#### 2.3.4 Glutaraldehyde concentration

Glutaraldehyde concentrations of (1.0, 2.0, 3.0, 4.0, and 5.0) wt% were evaluated using fixed concentrations of gelatine (5.0 wt%), hydrogen peroxide (3.6 wt%), and catalase (0.2 wt%). Glutaraldehyde was employed as a model cross-linker to enable precise analysis of structure–property relationships, rather than as a clinically finalised formulation component.

### 2.4 Foam characterisation

#### 2.4.1 Expansion ratio

Foam expansion was quantified immediately after solidification by measuring the foam height (*L*_foam_) with a plastic ruler. The initial foam height (*L*_foam,0_) was recorded immediately after apparent setting (within approximately 1–2 min of mixing), when the structure became self-supporting. The expansion ratio was calculated as:Expansion ratio = *L*_foam_/*L*_soln_where *L*_soln_ = 0.55 cm corresponds to the initial solution height for a 10 mL formulation.

#### 2.4.2 Foam stability (volume retention ratio)

Foam stability was assessed after 24 h at 37 °C by measuring the retained foam height (*L*_foam,24 h_). Stability was expressed as the volume retention ratio:*V*_24_/*V*_0_ = *L*_foam,24 h_/*L*_foam,0_

Values approaching unity indicate high structural stability. Relative volume changes were approximated from foam height assuming cylindrical geometry, which is justified by the uniform expansion observed within the cylindrical vessels.

### 2.5 Statistical analysis

Data are reported as mean ± SEM (*n* = 3) reflecting the uncertainty in the mean rather than population dispersion. Statistical analysis was performed using R (version 4.4.3). One-way ANOVA was applied to evaluate the effects of formulation parameters on foam expansion and stability, followed by Tukey's HSD post-hoc test where appropriate (*p* < 0.05). Full statistical outputs are provided in the SI.

## 3 Results and discussion

The development of advanced wound dressings requires materials that simultaneously provide efficient exudate absorption, maintain structural integrity under hydrated conditions, and conform to complex wound geometries.^1,2,15^ While hydrogel- and foam-based dressings have been widely explored for these purposes,^12,14,37,48^ many reported systems rely on mechanical foaming or chemical gas-forming agents that offer limited control over gas evolution kinetics and matrix stabilisation, often resulting in poor volume retention or inconsistent pore structures.^16,39,44^ In contrast, enzymatic foaming provides a chemically well-defined route to *in situ* gas generation under mild conditions, enabling close coupling between gas-generation dynamics and material structure.^47,49^

In the current system, foam formation is driven by catalase-mediated decomposition of hydrogen peroxide into water and molecular oxygen.^42^ The rate of oxygen generation is therefore expected to depend on classical enzyme kinetics and substrate availability. At the same time, the final foam morphology and stability are controlled by the physicochemical properties of the gelatine matrix and the extent of covalent cross-linking. Similar gas-expanded polymer systems have shown that the balance between gas generation, matrix viscosity, and network formation critically determines pore growth, coalescence, and collapse.^16,17,45^ The present results are consistent with established principles for enzymatically generated gelatine foams, in which formulation parameters can modulate expansion and stability.

Gelatine plays a dual chemical role in this system. As a biopolymer rich in amphiphilic amino acid residues, gelatine adsorbs at gas–liquid interfaces and lowers interfacial tension, stabilising oxygen bubbles during foam formation.^39–41,51^ Simultaneously, gelatine provides the reactive amine groups required for covalent cross-linking with glutaraldehyde. The observed dependence of foam expansion and volume retention on gelatine concentration is therefore consistent with the competing influences of interfacial stabilisation, solution viscosity, and network density. These trends have been reported for both gelatinous foams and gas-expanded hydrogels.^16,39,44^

Covalent stabilisation is achieved through glutaraldehyde-mediated formation of Schiff base linkages between aldehyde groups and primary amines on lysine residues within the gelatine chains.^43^ This reaction is expected to increase cross-link density and elastic modulus, suppressing bubble coalescence and gravitational collapse. However, excessive cross-linking may increase matrix rigidity and limit polymer chain mobility, thereby potentially hindering bubble growth during the early stages of oxygen evolution. Similar trade-offs between expansion and stability have been reported in chemically cross-linked gelatine films and hydrogel networks,^43,45,51^ supporting the interpretation of the trends observed in the present system.

From a functional perspective, foam stability over 24 h is a critical parameter for wound-dressing applications, as significant volume loss would compromise wound contact, moisture regulation, and exudate uptake.^5,11,29,46^ The ability of enzymatically generated foams to retain volume while maintaining high initial expansion highlights the potential advantages of this approach compared with mechanically foamed or purely chemically gas-blown materials, which often collapse rapidly in aqueous environments.^12,16,44^ The combination of enzymatic gas generation, interfacial stabilisation by gelatine, and covalent network fixation therefore provides a promising materials-based strategy for the design of flexible, absorbent, and shape-adaptive wound-dressing materials.

### 3.1 Effect of gelatine concentration on foam properties

#### 3.1.1 Foam expansion ratio

**Fig. 2:**
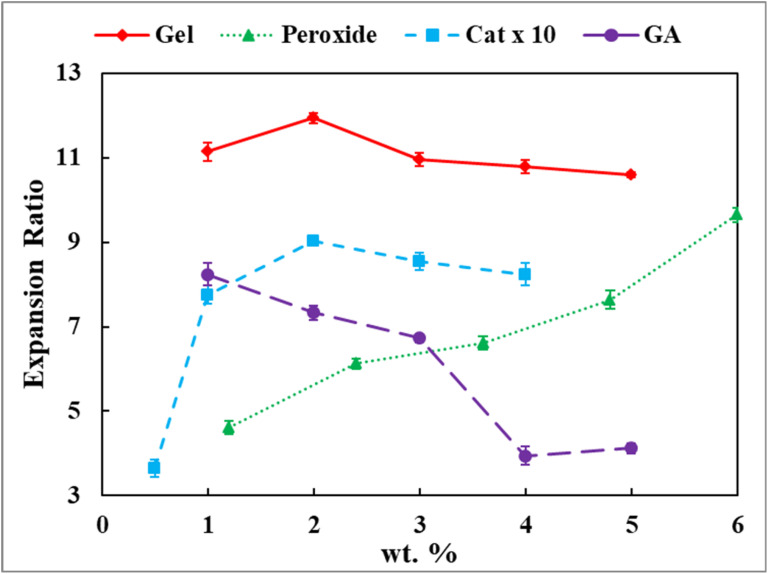
Expansion ratio of gelatine-based solidified foams as a function of formulation parameters. The expansion ratio (*L*_foam_/*L*_soln_) was calculated using an initial solution height of *L*_soln_ = 0.55 cm for a 10 mL formulation. Data are presented as mean ± SEM (*n* = 3). Data series are colour-coded and distinguished by markers: gelatine (red ◆), hydrogen peroxide (green ▲), catalase (blue ■; concentrations displayed as actual values × 10 for visual comparison), and glutaraldehyde (purple ●).

Foam expansion was evaluated at gelatine concentrations of (1.0, 2.0, 3.0, 4.0, and 5.0) wt% with fixed hydrogen peroxide (3.0 wt%), catalase (0.2 wt%), and glutaraldehyde (2.5 wt%). A statistically significant dependence of expansion ratio on gelatine concentration was observed (ANOVA, *F*(4, 10) = 11.51, *p* < 0.001; Table S2, SI), with 2.0 wt% gelatine yielding the highest expansion (Table 1 and Fig. 2). Tukey's HSD analysis confirmed that expansion at 2.0 wt% was significantly greater than at all other concentrations tested (Table S3, SI).

**Table 1:** Expansion ratio (*L*_foam_/*L*_soln_) of gelatine-based solidified foams at statistically relevant formulation levels. Expansion ratios were calculated using an initial solution height (*L*_soln_) of 0.55 cm, corresponding to a 10 mL formulation. Values are reported as mean ± SEM (*n* = 3). Complete datasets and pairwise Tukey HSD comparisons are provided in SI

Parameter varied	Concentration (wt%)	Expansion ratio (mean ± SEM)	Statistical outcome
Gelatine	2	Peak value (see Fig. 2)	Significantly higher than all other levels (ANOVA, *p* < 0.001)
Hydrogen peroxide	3.6	Plateau region (no significant difference *vs.* 2.4 wt%)	Enzyme saturation observed
Catalase	0.2	9.03 ± 0.06	Highest expansion (ANOVA, *p* < 0.001)
Glutaraldehyde	1	Highest expansion among GA levels	Significantly higher than ≥3.0 wt% (ANOVA, *p* < 0.001)

The non-monotonic expansion behaviour is consistent with the competing physicochemical roles of gelatine in enzymatically generated foams. Gelatine acts as a surface-active biopolymer that adsorbs at gas–liquid interfaces, reduces interfacial tension, and stabilises oxygen bubbles during catalase-mediated hydrogen peroxide decomposition.^39–41,51^ At low gelatine content (1.0 wt%), interfacial coverage and matrix viscosity appear insufficient to stabilise rapidly evolving oxygen bubbles, leading to coalescence and partial collapse, consistent with observations in weakly stabilised gelatine foams and gas-expanded hydrogels.^39,44^

At 2.0 wt% gelatine, a favourable balance is achieved between interfacial stabilisation, solution viscosity, and reaction kinetics. Oxygen generation *via* catalase^42^ proceeds at a rate that allows effective bubble growth before glutaraldehyde-mediated network fixation occurs.^43^ Similar synchronisation between gas evolution and matrix formation has been reported to be important for maximising expansion in CO_2_-foamed polymer systems.^16,17^

Further increases in gelatine concentration (≥3.0 wt%) result in reduced expansion. This behaviour is likely associated with increased solution viscosity and early network formation, which restricts bubble growth and oxygen diffusion through the matrix.^43–45^ Comparable viscosity-limited expansion has been reported for gelatinous foams and gas-expanded hydrogels at high polymer loadings.^16,39,44^ These findings indicate that gelatine concentration influences foam expansion by balancing interfacial chemistry, mass transport, and gelation kinetics.

#### 3.1.2 Foam stability (volume retention ratio, *V*_24_/*V*_0_)

**Fig. 3:**
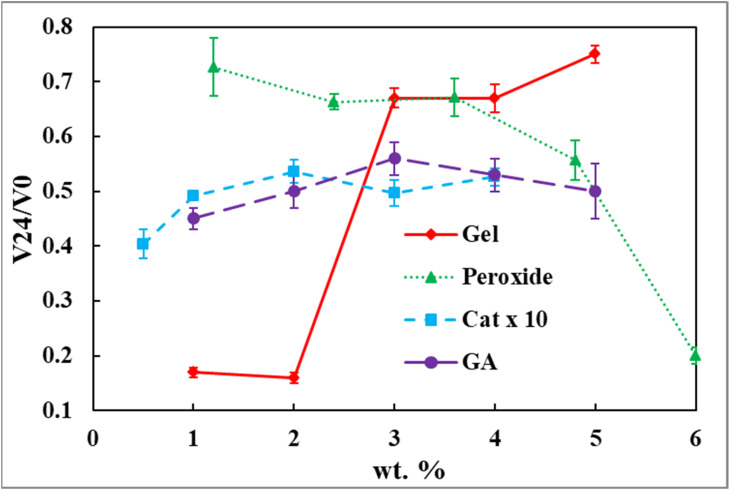
Stability of gelatine-based solidified foams after 24 h as a function of formulation parameters. Foam stability is expressed as the volume-retention ratio (*V*_24_/*V*_0_) defined as *L*_foam,24 h_/*L*_foam,0_. Values approaching unity indicate high structural stability. Data are presented as mean ± SEM from triplicate experiments (*n* = 3). Data series are colour-coded and distinguished by markers: gelatine (red ◆), hydrogen peroxide (green ▲), catalase (blue ■; concentrations displayed as actual values × 10 for visual comparison), and glutaraldehyde (purple ●).

Foam stability was quantified *via* the volume retention ratio after 24 h at 37 °C. Gelatine concentration exerted a highly significant effect on stability (ANOVA, *F*(4, 10) = 299.93, *p* < 0.001; Table S5, SI). Foams prepared with 3.0–5.0 wt% gelatine exhibited significantly higher volume retention than those prepared with 1.0 or 2.0 wt% (Table S6 and Fig. 3).

The enhanced stability at higher gelatine concentrations is consistent with increased cross-link density within the gelatine network. Glutaraldehyde reacts with primary amine groups on lysine residues to form Schiff base linkages, generating a covalently cross-linked three-dimensional network.^43^ Higher gelatine content increases the availability of reactive sites, producing a denser and mechanically stronger matrix that resists capillary-driven collapse, drainage, and bubble coalescence.^17,45,51^ Similar stabilisation effects have been reported in chemically cross-linked gelatine films and hydrogel scaffolds.^43,51^

In contrast, foams formed at 1.0–2.0 wt% gelatine exhibits lower network connectivity, rendering it more susceptible to surface-tension-driven thinning and moisture-induced deformation, resulting in significant volume loss over time.^45,51^ These trends align with prior studies on hydrocolloid- and gelatin-based dressings, in which polymer concentration is a primary determinant of long-term structural integrity under hydrated conditions.^14,40,41^

Notably, the gelatine concentration that maximises expansion (2.0 wt%) differs from that which maximises stability (3.0–5.0) wt%, highlighting a fundamental trade-off between bubble growth and network reinforcement. While lower gelatine concentrations favour expansion through reduced viscosity and delayed gelation,^39,44^ higher concentrations promote stability *via* increased cross-link density at the expense of expansion.^16,43^ Such decoupling of expansion and stability is characteristic of gas-expanded polymer systems and underscores the need for application-specific formulation design based on performance requirements.^16,17^

For wound-dressing applications requiring both initial porosity for exudate uptake and sustained structural integrity in moist environments,^4,12^ gelatine concentration therefore represents a critical design parameter.^40,41,50^ While 2.0 wt% gelatine offers maximal expansion, formulations containing (3.0–5.0) wt% gelatine provides superior long-term stability, suggesting greater suitability for prolonged wound contact under dynamic physiological conditions.

### 3.2 Effect of hydrogen peroxide concentration on foam properties

#### 3.2.1 Foam expansion ratio

Foam expansion was investigated at hydrogen peroxide concentrations of (1.2, 2.4, 3.6, 4.8 and 6.0) wt% using fixed gelatine (5.0 wt%), catalase (0.2 wt%), and glutaraldehyde (2.5 wt%) at 37 °C. Hydrogen peroxide concentration exerted a highly significant effect on expansion ratio (ANOVA, *F*(4, 10) = 124.62, *p* < 0.001; Table S8, SI), with expansion generally increasing with increasing hydrogen peroxide concentration (Table S7 and Fig. 2).

This behaviour is consistent with hydrogen peroxide's central role as the substrate for catalase-mediated oxygen generation. Increasing hydrogen peroxide concentration is expected to increase the rate of oxygen evolution, driving volumetric expansion of the gelatine matrix, consistent with classical enzyme kinetics and prior studies of gas-expanded polymer systems.^16,17,42^ However, Tukey's HSD analysis revealed no statistically significant difference between (2.4 and 3.6) wt% hydrogen peroxide (Table S9, SI), despite a numerical increase in expansion. This plateau may reflect the onset of catalase saturation, where the reaction rate approaches *V*_max_ and becomes independent of further increases in substrate concentration.^42^

Beyond enzyme kinetics, expansion is further moderated by the physicochemical constraints imposed by the polymeric matrix. The fixed gelatine–glutaraldehyde network exhibits finite viscosity and cross-link density, which can limit hydrogen peroxide diffusion to catalase active sites and restrict oxygen bubble growth at high gas-generation rates.^39,43–45^ A similar diffusion- and viscosity-limited expansion has been reported in gas-foamed hydrogels and gelatinous systems, where increasing the gas-generation rate does not translate linearly into increased expansion.^16,39,44^

At higher hydrogen peroxide concentrations (4.8–6.0) wt%, expansion increased significantly, indicating that oxygen generation may be approaching the matrix's stabilisation threshold. While such high expansion may be advantageous for creating highly porous structures, excessive gas evolution can compromise structural integrity, as discussed below. Control of hydrogen peroxide concentration, therefore, provides a direct chemical handle for tuning pore size and expansion, a principle widely recognised in gas-expanded biomaterials.^17,48^

#### 3.2.2 Foam stability (volume retention ratio, *V*_24_/*V*_0_)

Foam stability was assessed after 24 h at 37 °C by measuring the volume retention ratio (*V*_24_/*V*_0_). Hydrogen peroxide concentration had a highly significant effect on stability (ANOVA, *F* = 38.67, *p* = 4.69 × 10^−6^; Table S11, SI). Volume retention decreased progressively with increasing hydrogen peroxide concentration, falling from 0.73 at 1.2 wt% to 0.20 at 6.0 wt% (Table S10 and Fig. 3).

At low to moderate hydrogen peroxide concentrations (1.2–3.6) wt%, foam stability remained relatively high and statistically indistinguishable, indicating a favourable balance between oxygen-generation, interfacial stabilisation by gelatine, and glutaraldehyde-mediated network fixation. Under these conditions, oxygen evolution occurs at a rate that allows sufficient time for bubble stabilisation and covalent cross-linking, resulting in robust, volume-stable foams. Comparable stability windows have been reported in superporous hydrogels and enzymatically generated foams where gas evolution and gelation kinetics are well matched.^12,16,17^

In contrast, a pronounced decline in stability was observed at ≥4.8 wt% hydrogen peroxide, with 6.0 wt% exhibiting catastrophic volume loss. Tukey's analysis confirmed that foams prepared at 6.0 wt% were significantly less stable than all other formulations (Table S12, SI). This behaviour is consistent with a kinetic mismatch in which rapid oxygen generation may overwhelm the stabilisation capacity of the fixed gelatine (5.0 wt%) and glutaraldehyde (2.5 wt%) concentrations. Excessive internal gas pressure promotes bubble coalescence, film thinning, and rupture before sufficient cross-linking can occur, leading to structural collapse.^44,45^

This inverse relationship between gas-generation rate and long-term stability is a well-established principle in foam chemistry and polymer physics.^39,45^ Similar destabilisation at high gas-evolution rates has been reported for chemically foamed hydrogels and gas-expanded scaffolds, underscoring the need to control reaction kinetics rather than maximise gas output.^12,16^

Overall, these results indicate that hydrogen peroxide concentration strongly influences the balance between expansion and stability in enzymatically generated gelatine foams, concentrations in the range of (1.2–3.6) wt% provide a robust compromise between sufficient oxygen generation for expansion and controlled network formation for long-term stability, making them most suitable for wound-dressing applications requiring sustained contact, moisture retention, and exudate absorption under physiological conditions.^4,11,29^

### 3.3 Effect of catalase concentration on foam properties

#### 3.3.1 Foam expansion ratio

The effect of catalase concentration on foam expansion was evaluated using (0.05, 0.10, 0.20, 0.30, and 0.40) wt% while maintaining fixed gelatine (5.0 wt%), hydrogen peroxide (3.6 wt%), and glutaraldehyde (2.5 wt%) concentrations. Catalase concentration had a highly significant effect on foam expansion (ANOVA, *F*(4, 10) = 113.03, *p* < 2.8 × 10^−8^; Table S14, SI). The expansion ratio increased sharply from 3.64 ± 0.21 at 0.05 wt% catalase to a maximum of 9.03 ± 0.06 at 0.20 wt%, followed by a modest, statistically non-significant decline at higher enzyme concentrations (Table S13 and Fig. 2).

This non-linear trend reflects the kinetic interplay between enzymatic gas generation and matrix solidification. Catalase controls the rate of hydrogen peroxide decomposition into oxygen. At very low enzyme concentrations, the rate of gas evolution is insufficient to expand the gelatine matrix before glutaraldehyde-mediated cross-linking increases viscosity and arrests bubble growth. Similar gas-limited expansion regimes have been reported in enzyme-driven foams and gas-expanded hydrogels, where early gelation suppresses pore development.^16,17^

At 0.20 wt% catalase, oxygen generation proceeds at a rate that is optimally matched to gelatine interfacial stabilisation and network formation, enabling efficient bubble growth and entrapment before matrix fixation. This kinetic synchronisation between gas evolution and gelation has been identified as a key requirement for maximising expansion in both chemically and enzymatically foamed polymer systems.^16,39,44^

Further increases in catalase concentration (≥0.30 wt%) lead to a slight reduction in expansion. At these levels, rapid oxygen generation can locally exceed the stabilisation capacity of the forming gel network, promoting bubble coalescence and premature rupture before complete cross-linking occurs. Comparable over-foaming effects have been observed in fast-reacting gas-forming systems, where excessive gas flux reduces final expansion despite higher theoretical gas yields.^44,45^ These results highlight the need to control enzyme concentration to balance gas-generation kinetics with matrix solidification.

#### 3.3.2 Foam stability (volume retention ratio, *V*_24_/*V*_0_)

Foam stability was assessed after 24 h at 37 °C using the volume retention ratio (*V*_24_/*V*_0_). Catalase concentration exerted a statistically significant effect on stability (ANOVA, *F*(4, 10) = 6.5845, *p* = 0.0073; Table S17, SI). Tukey's HSD analysis revealed that foams prepared with 0.20 and 0.40 wt% catalase exhibited significantly higher volume retention than those prepared with 0.05 wt% catalase, while no significant differences were observed among intermediate concentrations (Table S18, SI).

The increase in stability with catalase concentration reflects improved temporal coordination between gas evolution and network formation. At low catalase concentration (0.05 wt%), slow oxygen generation produces large, poorly stabilised bubbles that are vulnerable to drainage and collapse before sufficient glutaraldehyde cross-linking occurs. In contrast, higher catalase concentrations promote more rapid and spatially uniform bubble nucleation, allowing gelatine adsorption at gas–liquid interfaces and timely covalent fixation of the structure.^39,43^

The stabilisation plateau observed beyond 0.20 wt% catalase indicates that the system transitions from enzyme-limited to substrate- or matrix-limited behaviour. Once hydrogen peroxide availability or cross-linkable amine density becomes limiting, further increases in enzyme concentration do not enhance structural integrity. Such saturation behaviour is consistent with enzyme kinetics and has been reported in other enzymatically mediated polymer systems.^41^ Similarly, the extent of network reinforcement is ultimately constrained by gelatine concentration and glutaraldehyde availability.^40,43^

From an application perspective, the combination of high expansion and stable volume retention at intermediate catalase concentrations is particularly relevant for wound-dressing materials. Stable foams are better able to maintain wound contact, regulate moisture, absorb exudate, and facilitate oxygen transport over extended periods.^4,5,46^ The results demonstrate that catalase concentration provides a powerful kinetic control parameter for tuning both the foam architecture and the long-term stability of enzymatically generated gelatine foams.

### 3.4 Effect of glutaraldehyde concentration on foam properties

#### 3.4.1 Foam expansion ratio

Foam expansion was evaluated as a function of glutaraldehyde (GA), used as a model cross-linking agent, at concentrations of 1.0, 2.0, 3.0, 4.0, and 5.0 wt%. All formulations were prepared using fixed concentrations of gelatine (5.0 wt%), hydrogen peroxide (3.6 wt%), and catalase (0.2 wt%) and processed at 37 °C. Glutaraldehyde concentration exerted a highly significant effect on foam expansion (ANOVA, *F*(4, 10) = 111.6, *p* = 2.97 × 10^−8^; Table S20, SI). The highest expansion ratios were obtained at low GA concentrations, with expansion decreasing progressively as GA concentration increased (Table S19 and Fig. 2).

This trend reflects the dual chemical role of glutaraldehyde as a network-forming agent and viscosity modifier. GA reacts with primary amine groups on lysine and hydroxylysine residues in gelatine to form Schiff base (imine) linkages, producing a covalently cross-linked network [43, 52]. At very low GA concentration (1.0 wt%), cross-link density is insufficient to mechanically stabilise the rapidly expanding oxygen bubbles, resulting in partial collapse despite high apparent expansion. Similar instability at low cross-linker content has been reported in gelatinous foams and chemically cross-linked hydrogels.^43,45^

At intermediate GA concentration (≈2.0 wt%), a robust balance is achieved between network formation and chain mobility. Sufficient cross-linking stabilises the gas–liquid interfaces without excessively increasing viscosity, allowing efficient bubble growth and fixation. This “sweet-spot” behaviour has been widely observed in gas-expanded polymer systems, where moderate cross-link density maximises expansion by synchronising gas evolution with gelation kinetics.^16,17,44^

Further increases in GA concentration (≥3.0 wt%) lead to a marked reduction in expansion. At these levels, rapid cross-linking increases matrix rigidity and viscosity, limiting polymer chain rearrangement and physically hindering bubble growth and coalescence.^16,44^ Restricted gas diffusion and premature network fixation produce smaller, less expandable pores, consistent with reports on highly cross-linked gelatine films and hydrogel scaffolds.^43,45,51^ These results demonstrate that GA concentration strongly influences foam expansion by controlling cross-link density and viscoelastic resistance.

#### 3.4.2 Foam stability (volume retention ratio, *V*_24_/*V*_0_)

Foam stability was assessed after 24 h at 37 °C using the volume retention ratio (*V*_24_/*V*_0_). Although mean stability increased from 0.45 ± 0.02 at 1.0 wt% GA to a maximum of 0.56 ± 0.03 at 3.0 wt% GA (Table S22 and Fig. 3), a one-way ANOVA did not identify a statistically significant overall effect of GA concentration on stability (*F*(4, 10) = 1.73, *p* = 0.219; Table S23, SI).

Despite the absence of statistical significance, the observed trend is chemically meaningful and consistent with the mechanism of GA cross-linking. Increasing GA concentration increases the density of Schiff base linkages within the gelatine network, enhancing elastic modulus and resistance to capillary-driven collapse, drainage, and gas diffusion [43, 52–55]. At low GA concentration (1.0 wt%), the sparse network lacks sufficient mechanical integrity to counteract internal stresses generated during foaming, resulting in pronounced volume loss over time.

A numerical maximum was observed at ∼3.0 wt% GA; however, differences were not statistically significant. This trend is consistent with the formation of a sufficiently dense, interconnected network capable of maintaining foam architecture under hydrated conditions. However, no statistically significant dependence of foam stability on glutaraldehyde concentration was observed. Similar stabilisation plateaus have been reported in gelatinous hydrogels and chemically cross-linked scaffolds, where additional cross-linking beyond a critical density yields diminishing improvements in macroscopic stability.^43,51^ At higher GA concentrations, further increases in cross-link density do not significantly enhance volume retention, as network reinforcement becomes limited by polymer concentration rather than cross-linker availability.

Comparison of expansion and stability trends highlights a fundamental trade-off inherent to gas-expanded polymer systems. Conditions that favour maximal expansion (low GA, low viscosity) yield poor long-term stability, whereas higher cross-link densities improve stability at the expense of expansion. This balance between foamability and mechanical reinforcement is a central design principle in hydrogel and foam-based biomaterials.^4,12,16^

For wound-dressing applications requiring sustained contact, moisture retention, and mechanical integrity, intermediate GA concentrations provide the most favourable compromise between initial porosity and long-term structural stability.^40,41,46^ These findings underscore the importance of cross-linking chemistry as a key determinant of structure–property relationships in enzymatically generated gelatine foams.

### 3.5 Synthesis of formulation–structure relationships and integrated optimisation window

Collectively, the parametric optimisation demonstrates that the kinetic balance between catalase-mediated oxygen evolution, interfacial stabilisation by gelatine, and covalent network formation *via* glutaraldehyde cross-linking plays a central role in determining foam expansion and long-term stability in enzymatically generated gelatine foams. Gelatine concentration controls interfacial activity, viscosity, and cross-linkable amine density, leading to distinct optima for expansion and stability, consistent with trends reported for gas-expanded hydrogels and gelatinous foams.^16,39,44^ Hydrogen peroxide concentration dictates oxygen generation rate according to enzyme kinetics, where excessive gas flux destabilises the matrix despite increased expansion, a behaviour well documented in superporous hydrogel systems.^12,16^ Catalase concentration modulates the temporal synchronisation between gas evolution and gelation. In contrast, glutaraldehyde concentration strongly influences cross-link density and viscoelastic resistance, together defining the fundamental expansion-stability trade-off characteristic of chemically cross-linked polymer networks.^43,51^

Integration of expansion and stability data (Fig. 2, 3 and Tables 1, 2) identifies a well-defined formulation window (2.0–3.0 wt% gelatine, 3.0–4.0 wt% hydrogen peroxide, 0.2 wt% catalase, and 2.5–3.0 wt% glutaraldehyde) in which sufficient oxygen generation enables rapid expansion while timely covalent cross-linking preserves structural integrity.

**Table 2:** Volume retention ratio (*V*_24_/*V*_0_) of gelatine-based solidified foams after 24 h, at statistically relevant formulation levels. Foam stability was defined as *V*_24_/*V*_0_ = *L*_foam,24 h_/*L*_foam,0_. Values are reported as mean ± SEM (*n* = 3). Full statistical outputs are provided in the SI

Parameter varied	Concentration (wt%)	*V*_24_/*V*_0_ (mean ± SEM)	Statistical outcome
Gelatine	5	High retention (see Fig. 3)	Significantly higher than ≤2.0 wt% (one-way ANOVA, *p* < 0.001)
Hydrogen peroxide	6	0.20 ± SEM	Significantly lower than all other levels (one-way ANOVA, *p* < 0.001)
Catalase	0.2	Improved stability plateau	Significant *vs.* 0.05 wt% (*p* = 0.001)
Glutaraldehyde	3	0.56 ± 0.03	Highest mean value; not statistically significant

**Fig. 4:**
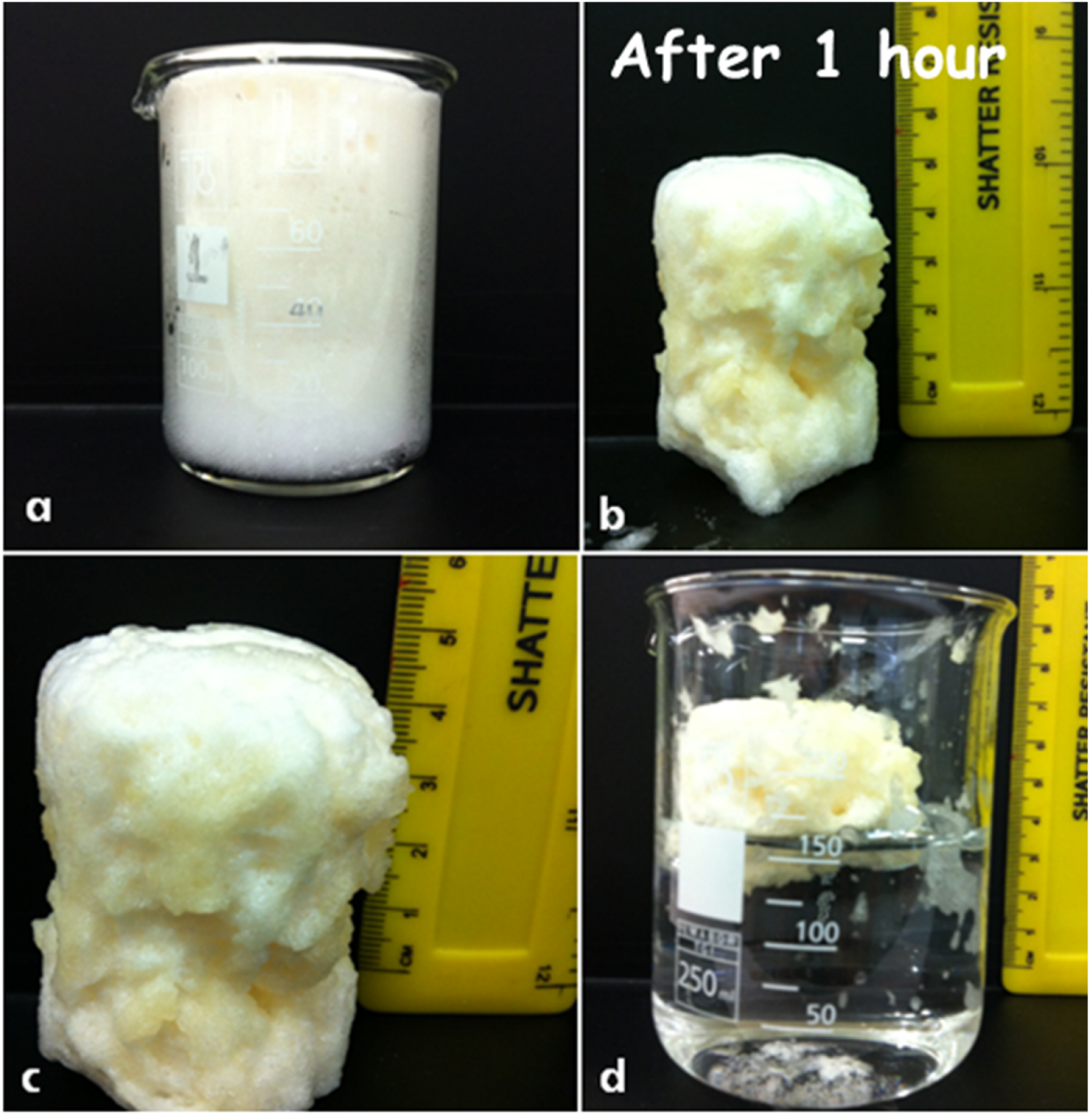
Optimised solidified gelatine foam system formed by adding 2 mL of solution B (GA 2.5 wt%, catalase 0.2 wt%) to 8 mL of solution A (gelatine 3.0 wt%, hydrogen peroxide 3.6 wt%) at 37 °C. The figure shows that the solidified foam conforms to the beaker's shape, suggesting its ability to adapt to wound contours. Additionally, the foam floats on water, indicating its potential for prolonged absorption of wound exudates.

A representative foam prepared within this formulation window is shown in Fig. 4. The foam readily conforms to complex geometries, such as beaker walls, and remains buoyant on water, demonstrating properties directly relevant to wound contour adaptation and prolonged fluid absorption. Foams produced within this window exhibit high expansion ratios, sustained volume retention, and mechanical flexibility. Collectively, these characteristics support their suitability for biomedical foam applications requiring conformability and prolonged functional performance, such as wound dressings. In contrast to previously reported gelatine-based foams produced by mechanical or chemical foaming routes, the present enzymatic system enables rapid *in situ* expansion and stabilisation under physiologically relevant conditions while providing systematic chemical control over expansion–stability trade-offs.^16,17,39^

#### 3.5.1 Limitations and scope

While the present study establishes clear formulation–structure relationships for enzymatically generated gelatine foams, several limitations should be acknowledged. Foam characterisation was based primarily on macroscopic expansion and 24 h volume retention measurements, which provide functionally relevant metrics for wound-dressing performance but do not directly resolve pore-scale morphology, rheological behaviour, or cross-link density. In addition, although foam setting occurred rapidly under all conditions, detailed time-resolved monitoring of post-expansion height evolution was beyond the scope of the current formulation-focused study. The use of glutaraldehyde as a model cross-linker enabled controlled interrogation of structure–property relationships; however, evaluating clinically preferred cross-linking chemistries and quantifying residual cross-linker content will be required for translational applications. Future work will therefore focus on microstructural imaging, mechanical characterisation, and wound-mimetic testing to further refine the physicochemical understanding and biomedical readiness of this enzymatically generated foam platform.

## 4 Conclusions

This work presents a systematic physicochemical optimisation of enzymatically generated, gelatine-based solidified foam formed *via* catalase-mediated oxygen evolution and covalent network fixation. By independently varying the concentrations of gelatine, hydrogen peroxide, catalase, and glutaraldehyde, the coupled roles of enzymatic gas generation, interfacial stabilisation, and cross-linking chemistry were elucidated under physiologically relevant conditions. The results demonstrate that foam expansion and long-term stability are strongly influenced by kinetic synchronisation between oxygen evolution and matrix stiffening, rather than by any single formulation parameter.

Maximum expansion was achieved at intermediate gelatine and catalase concentrations, where surface activity, viscosity, and gas-generation rate were favourably balanced. In contrast, excessive hydrogen peroxide concentrations destabilised the foam by producing oxygen at rates that likely exceeded the stabilisation and cross-linking capacity of the gelatine–glutaraldehyde network, consistent with enzyme saturation and mass-transport limitations. Increasing gelatine content enhanced long-term stability through increased cross-link density, while glutaraldehyde concentration modulated the trade-off between initial foamability and network rigidity.

Integration of expansion and stability data identified a robust formulation window (2.0–3.0 wt% gelatine, 3.0–4.0 wt% hydrogen peroxide, 0.2 wt% catalase, and 2.5–3.0 wt% glutaraldehyde), providing a balanced compromise between rapid expansion and sustained 24 h volume retention.

Compared with mechanically or chemically foamed gelatine systems, the present enzymatic approach enables rapid *in situ* expansion and stabilisation under mild conditions while offering systematic chemical control over expansion–stability trade-offs.^16,17,40,41^ Importantly, the observed trends are consistent with fundamental chemical processes (enzyme kinetics, gas–liquid mass transport, interfacial stabilisation, and covalent network formation), placing this work firmly within chemistry-driven materials design rather than empirical formulation screening.

In this study, glutaraldehyde was deliberately employed as a model cross-linker to enable precise examination of formulation–structure relationships. Building on this framework, ongoing work is focused on extending the platform to support clinically relevant cross-linking strategies, investigating pH-responsive behaviour in chronic wound environments, and quantifying fluid-management performance using simulated wound exudate. Future studies will also explore the incorporation of therapeutic agents and evaluation under wound-mimetic conditions, further advancing the translational potential of enzymatically generated gelatin-based foam dressings.

## Conflicts of interest

There are no conflicts to declare.

## Supplementary Material

RA-016-D6RA00030D-s001

RA-016-D6RA00030D-s002

## Data Availability

The data supporting the findings of this study was collected by Shwan Abdullah Hamad and has not been published in any form. Supplementary information (SI) is available. See DOI: https://doi.org/10.1039/d6ra00030d.
