# Supplementary material for: Gelatine-based foams produced by enzymatic foaming: formulation–structure relationships affecting expansion and stability
Source: RSC Adv. 2026 Mar 2;16(13):11944–54. doi: 10.1039/d6ra00030d (PMC12951601; doi:10.1039/d6ra00030d)
Supplement: RA-016-D6RA00030D-s001 [file RA-016-D6RA00030D-s001.pdf]

Supplementary Information (SI) for RSC Advances.
This journal is © The Royal Society of Chemistry 2026

Electronic Supplementary Information (ESI)

Supplementary data support the formulation–structure relationships discussed in the main manuscript.

This Electronic Supplementary Information provides detailed experimental data, statistical analyses, and additional figures supporting the formulation optimisation and structure–property relationships discussed in the main manuscript. The ESI includes raw measurements, calculated expansion ratios and volume retention ratios, and complete one-way ANOVA and Tukey HSD post hoc outputs for all formulation variables investigated. Foam expansion and structural fixation occurred within seconds under all conditions; therefore, L_0 was recorded immediately after visible cessation of macroscopic expansion.

Unless otherwise stated, foam formation was conducted under open-headspace conditions. In selected photographic figures, samples were briefly placed in loosely capped vials after initial foaming to minimise disturbance during imaging while maintaining internal headspace.

Foam expansion was quantified using height-based measurements and expressed as the expansion ratio (L_{foam}/L_{soln}), where L_{foam} is the foam height immediately after solidification and L_{soln} = 0.55 cm corresponds to the initial solution height for a 10 mL formulation. Foam stability was evaluated after 24 h at 37 °C and reported as the volume retention ratio ($V_{24}/V_0 = L_{foam,24\ h}/L_{foam,0}$). Height-based measurements were used as a practical approximation of relative volume changes, assuming approximately cylindrical geometry, which is justified by the uniform expansion observed within the cylindrical vessels.

Unless otherwise stated, all experiments were performed in triplicate (n = 3), and data are reported as mean ± standard error of the mean (SEM). Statistical significance was assessed using one-way ANOVA followed by Tukey's HSD post-hoc testing ($p < 0.05$).

1.1 Effect of Gelatine Concentration on the Expansion Ratio of Solidified Gelatine-Based Foam:

wt. % Gelatine	Vsoln = Lsoln	Vfoam-1	Vfoam-2	Vfoam-3	Expansion Ratio-1	Expansion Ratio-2	Expansion Ratio-3	Mean Expansion Ratio	SEM
1	0.55	6.3	5.9	6.2	11.45	10.73	11.27	11.15	0.22
2	0.55	6.5	6.7	6.5	11.82	12.18	11.82	11.94	0.12
3	0.55	6	6.2	5.9	10.91	11.27	10.73	10.97	0.16
4	0.55	5.9	5.8	6.1	10.73	10.55	11.09	10.79	0.16
5	0.55	5.8	5.9	5.8	10.55	10.73	10.55	10.61	0.06

Table S1: Expansion Ratio of Solidified Foam at Varying Gelatine Concentrations. Foam was synthesised with fixed concentrations of hydrogen peroxide (3 wt. %), catalase (0.2 wt. %), and glutaraldehyde (2.5 wt. %) in a 10 mL solution.

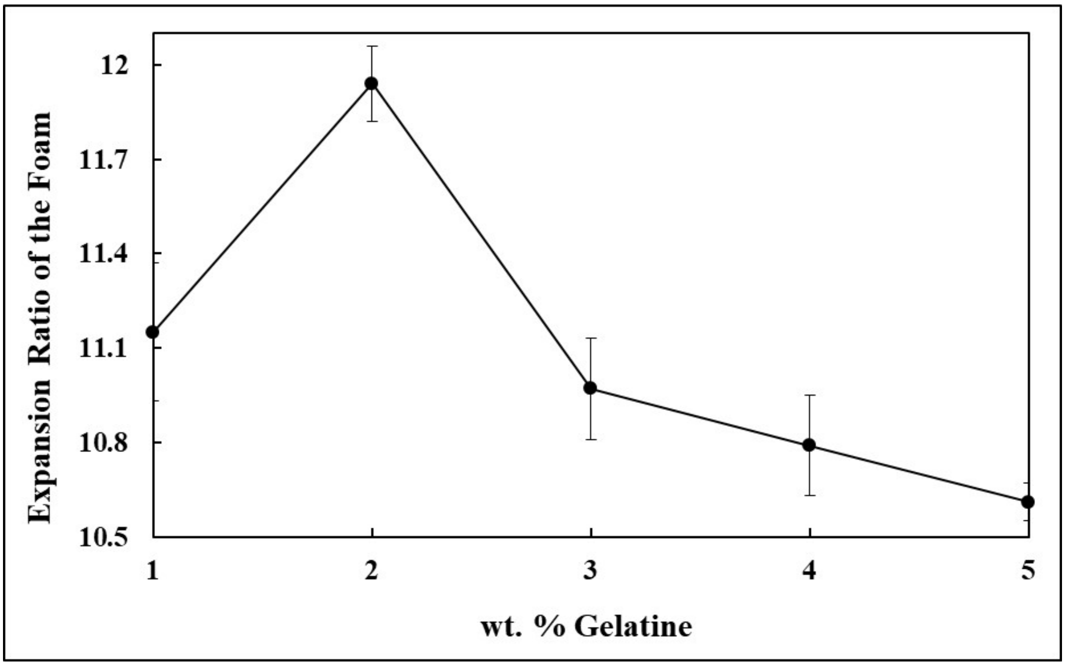

Figure S1: Expansion Ratio of Solidified Foam at Varying Gelatine Concentrations. Foam was synthesised with fixed concentrations of hydrogen peroxide (3 wt. %), catalase (0.2 wt. %), and glutaraldehyde (2.5 wt. %) in a 10 mL solution.

**Source of Variation**	**SS**	**df**	**MS**	**F**	**P-value**
Between Groups	3.18264	4	0.79566	11.5113	0.000923739
Within Groups	0.6912	10	0.06912		
Total	3.87384	14			

Table S2: One-Way ANOVA for Effect of Gelatine Concentration on Mean Expansion Ratio. A significant effect of gelatine concentration (1.0, 2.0, 3.0, 4.0, 5.0) wt. % on the mean foam expansion ratio was observed ($F(4, 10)= 11.511$, $p = 0.00092$, $p < 0.001$). Each group had three replicates ($n = 3$).

Comparison of Gelatine wt. %	Difference in Means	95 % Lower CI	95 % Upper CI	Adjusted p-value	Significance (α = 0.05)
2.0 - 1.0	0.79	0.08	1.5	0.027	Yes
3.0 - 1.0	-0.18	-0.89	0.53	0.912	No
4.0 - 1.0	-0.36	-1.07	0.35	0.487	No
5.0 - 1.0	-0.54	-1.25	0.17	0.163	No
3.0 - 2.0	-0.97	-1.68	-0.26	0.008	Yes
4.0 - 2.0	-1.15	-1.86	-0.44	0.002	Yes
5.0 - 2.0	-1.33	-2.04	-0.62	0.001	Yes
4.0 - 3.0	-0.18	-0.89	0.53	0.912	No
5.0 - 3.0	-0.36	-1.07	0.35	0.487	No
5.0 - 4.0	-0.18	-0.89	0.53	0.912	No

Table S3: Tukey's HSD Post-Hoc Analysis for Effect of Gelatine Concentration on Expansion Ratio. Pairwise comparisons revealed that 2.0 wt. % gelatine yields significantly higher expansion ratios than 1.0 wt. %, 3.0 wt. %, 4.0 wt. %, and 5.0 wt. % (adjusted $p < 0.05$). All other comparisons were non-significant.

1.2 Effect of Gelatine Concentration on the Stability (Volume Retention Ratio V_{24}/V_0) of Solidified Gelatine-Based Foam:

wt. % Gelatine	V0-1	V24-1	V24/V0 (1)	V0-2	V24-2	V24 / V0 (2)	V0-3	V24-3	V24/V0 (3)	Mean of V24/V0	SEM of V24/V0
1	6.3	1.1	0.17	5.9	1.1	0.19	6.2	1	0.16	0.17	0.0088
2	6.5	1	0.15	6.7	1	0.15	6.5	1.2	0.18	0.16	0.01
3	6	3.9	0.65	6.2	4.1	0.66	5.9	4.2	0.71	0.67	0.0177
4	5.9	3.7	0.63	5.8	4.2	0.72	6.1	4.1	0.67	0.67	0.026
5	5.8	4.5	0.78	5.9	4.3	0.73	5.8	4.3	0.74	0.75	0.0153

Table S4: Effect of Gelatine Concentration on the Stability of Solidified Gelatine-Based Foam. Stability is expressed as the volume retention ratio (V_{24}/V_0) with fixed concentrations of hydrogen peroxide (3 wt. %), catalase (0.2 wt. %), and glutaraldehyde (2.5 wt. %) in a 10 mL solution. Foam stability is expressed as the volume retention ratio (V_{24}/V_0).

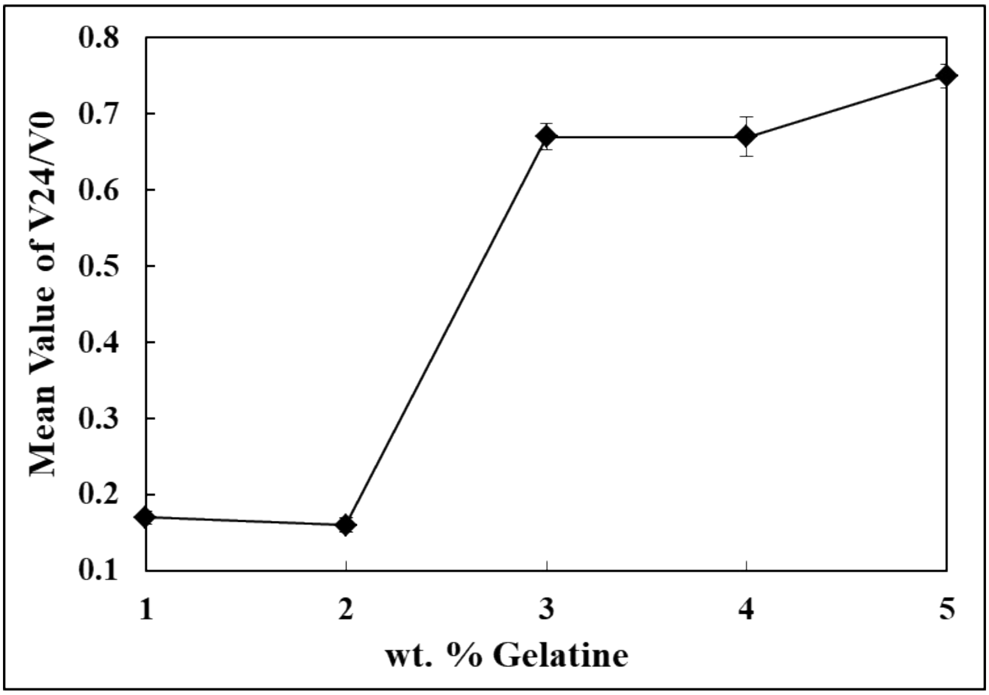

Figure S2: Effect of Gelatine Concentration on the Stability of Solidified Gelatine-Based Foam. Stability is expressed as the volume retention ratio (V_{24}/V_0) with fixed concentrations of hydrogen peroxide (3 wt. %), catalase (0.2 wt. %), and glutaraldehyde (2.5 wt. %) in a 10 mL solution. Mean volume retention ratios are presented with standard error of the mean (SEM) (n = 3).

**Source of Variation**	**SS**	**df**	**MS**	**F**	**P-value**
Between Groups	1.0318	4	0.25794	299.9	2.30E-10
Within Groups	0.0086	10	0.00086		
Total	1.0404	14			

Table S5: One-way ANOVA for Effect of Gelatine Concentration on Mean Volume Retention Ratio. Gelatine concentration (1.0, 2.0, 3.0, 4.0, and 5.0) wt. % had a statistically significant effect on the mean foam volume retention ratio ($F(4, 10)= 299.9$, $p = 2.3 \times 10^{-10}$, $p < 0.001$). Each group had three replicates (n = 3).

Comparison of Gelatine wt. %	Difference in Means	95 % Lower CI	95 % Upper CI	Adjusted p-value	Significance (α = 0.05)
2.0 - 1.0	-0.0133	-0.092	0.065	0.9785	No
3.0 - 1.0	0.5	0.421	0.579	0	Yes
4.0 - 1.0	0.5	0.421	0.579	0	Yes
5.0 - 1.0	0.5767	0.498	0.655	0	Yes
3.0 - 2.0	0.5133	0.435	0.592	0	Yes
4.0 - 2.0	0.5133	0.435	0.592	0	Yes
5.0 - 2.0	0.59	0.511	0.669	0	Yes
4.0 - 3.0	0	-0.079	0.079	1	No
5.0 - 3.0	0.0767	-0.002	0.155	0.0574	No
5.0 - 4.0	0.0767	-0.002	0.155	0.0574	No

Table S6: Tukey's HSD Post-Hoc Analysis for Effect of Gelatine Concentration on Stability (Volume Retention Ratio V24/V0). Comparisons showed gelatine concentrations of 3.0 wt. %, 4.0 wt. %, and 5.0 wt. % significantly improved foam stability (volume retention) compared to lower concentrations (adjusted $p < 0.05$).

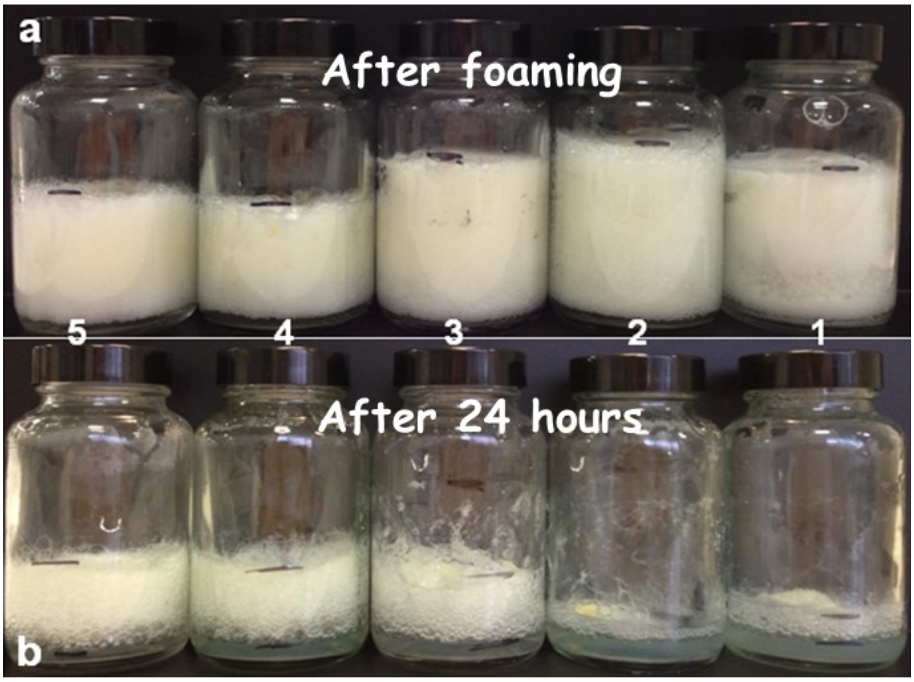

Figure S3: Solidified Foams at Varying Gelatine Concentrations. Photographs show foams immediately after formation (a) and after 24 hours (b), illustrating expansion and volume retention. Formed from 2.0 mL catalase/glutaraldehyde (0.2 wt. % Cat, 2.5 wt. % GA) mixed with 8.0 mL gelatine/hydrogen peroxide (3.0 wt. %) at 37 °C; gelatine concentration varied from 1.0 to 5.0 wt. %.

2.1 Effect of Hydrogen Peroxide Concentration on the Expansion Ratio of Solidified Gelatine-Based Foam:

wt. % Hydrogen Peroxide	Vsoln = Lsoln (cm)	Vo−1 = L0-1 (cm)	Vo−2 = Lo-2 (cm)	Vo−3 = L0-3 (cm)	Expansion Ratio-1	Expansion Ratio-2	Expansion Ratio-3	Mean Expansion Ratio	SEM
1.2	0.55	2.5	2.7	2.4	4.55	4.91	4.36	4.61	0.16
2.4	0.55	3.3	3.5	3.3	6	6.36	6	6.12	0.12
3.6	0.55	3.5	3.6	3.8	6.36	6.55	6.91	6.61	0.16
4.8	0.55	4.2	4.4	4	7.64	8	7.27	7.64	0.21
6	0.55	5.2	5.23	5.5	9.45	9.51	10	9.65	0.17

Table S7: Observed Volumes, Calculated Expansion Ratios, and Mean Expansion Ratios with Standard Error of the Mean for varying wt. % Hydrogen Peroxide concentrations. Experimental parameters: 10 mL final system volume, 5.0 wt. % gelatine, 0.2 wt. % catalase, 2.5 wt. % glutaraldehyde, 37 °C.

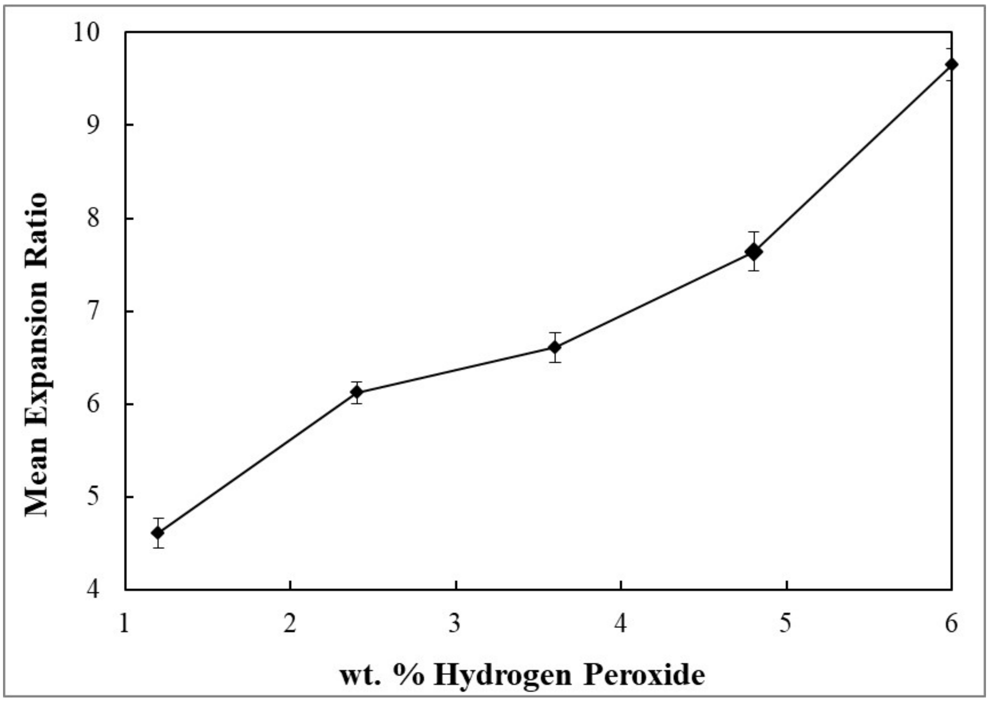

Figure S4: Mean Expansion Ratio versus wt. % Hydrogen Peroxide. Error bars represent SEM (n = 3). Fixed gelatine (5.0 wt. %), catalase (0.2 wt. %), glutaraldehyde (2.5 wt. %), 37 °C, 10 mL solution.

Source of Variation	Sum of Squares (SS)	Degrees of Freedom (df)	Mean Square (MS)	F-statistic	P-value
Between Groups	42.2229	4	10.5557	124.6151	1.74E-08
Within Groups	0.8471	10	0.0847		
Total	43.07	14			

Table S8: One-Way ANOVA for the effect of wt. % Hydrogen Peroxide Concentration on Mean Expansion Ratio. F(4, 10)= 124.62, p < 0.001, indicating a highly significant effect of hydrogen peroxide concentration on foam expansion (n = 3 per group).

Comparison of wt. % H_2O_2	Difference in Means	95% Confidence Interval (Lower)	95% Confidence Interval (Upper)	Adjusted p-value	Significant (α = 0.05)
2.4 – 1.2	1.5133	0.7313	2.2954	0.0006029	Yes
3.6 – 1.2	2	1.2179	2.7821	0.0000575	Yes
4.8 – 1.2	3.03	2.2479	3.8121	0.0000013	Yes
6.0 – 1.2	5.0467	4.2646	5.8287	< 0.0000001	Yes
3.6 – 2.4	0.4867	-0.2954	1.2687	0.3117275	No
4.8 – 2.4	1.5167	0.7346	2.2987	0.0005923	Yes
6.0 – 2.4	3.5333	2.7513	4.3154	0.0000003	Yes
4.8 – 3.6	1.03	0.2479	1.8121	0.0100681	Yes
6.0 – 3.6	3.0467	2.2646	3.8287	0.0000012	Yes
6.0 – 4.8	2.0167	1.2346	2.7987	0.0000534	Yes

Table S9: Tukey's HSD Post-Hoc Test Results for Pairwise Comparisons of Mean Expansion Ratios across different wt. % Hydrogen Peroxide concentrations. Notable: No statistically significant difference between 2.4 and 3.6 wt. % hydrogen peroxide (adjusted p = 0.311).

2.2 Effect of Hydrogen Peroxide Concentration on the Stability (Volume Retention Ratio V24/V0) of Solidified Gelatine-Based Foam:

wt. % Hydrogen Peroxide	Vo-1	Vo-2	Vo-3	V24-1	V24-2	V24-3	V24-1/Vo-1	V24-2/Vo-2	V24-3/Vo-3	Mean V24/Vo	SEM (V24/Vo)
1.2	2.5	2.7	2.4	1.7	1.8	2	0.68	0.6667	0.8333	0.7267	0.0535
2.4	3.3	3.5	3.3	2.2	2.4	2.1	0.6667	0.6857	0.6364	0.6629	0.0144
3.6	3.5	3.6	3.8	2.4	2.6	2.3	0.6857	0.7222	0.6053	0.6711	0.0345
4.8	4.2	4.4	4	2.1	2.4	2.5	0.5	0.5455	0.625	0.5568	0.0365
6	5.2	5.23	5.5	1	1.2	0.98	0.1923	0.2294	0.1782	0.2	0.0153

Table S10: Initial foam volume (Vo) and foam volume after 24 hours (V24) for triplicate experiments at varying hydrogen peroxide concentrations, along with individual mean volume retention ratios (V_{24}/V_o) and standard error of the mean (SEM). Experimental parameters: 10 mL final system volume, 5.0 wt. % gelatine, 0.2 wt. % catalase, 2.5 wt. % glutaraldehyde, 37 °C.

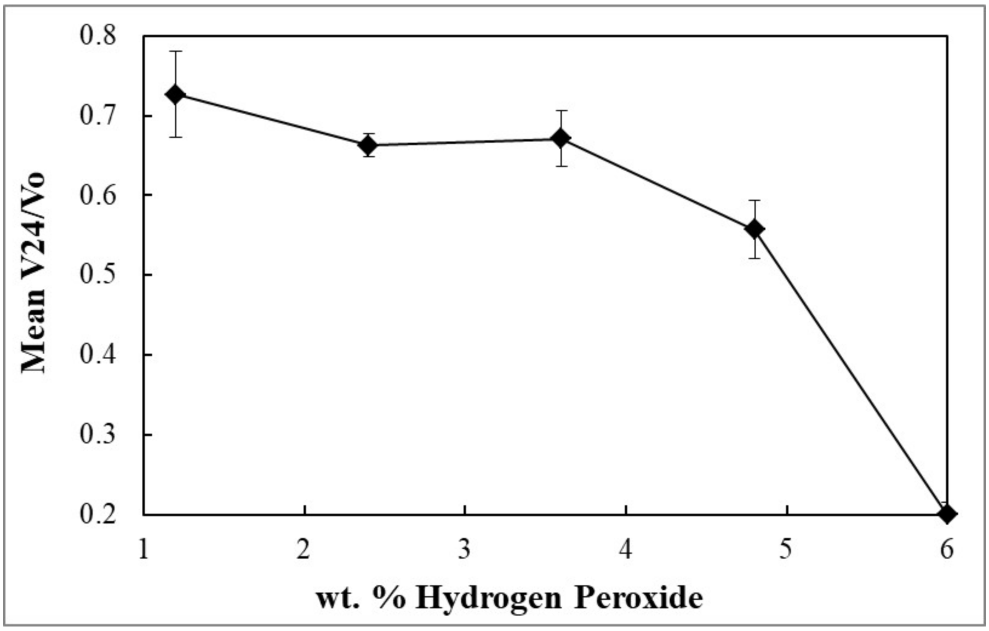

Figure S5: Mean volume retention ratio (V_{24}/V_o) versus wt. % Hydrogen Peroxide. Error bars represent SEM (n = 3). Fixed gelatine (5.0 wt. %), catalase (0.2 wt. %), glutaraldehyde (2.5 wt. %), 37 °C, 10 mL solution.

Source of Variation	Sum of Squares (SS)	Degrees of Freedom (df)	Mean Square (MS)	F-statistic	P-value
**Between Groups**	0.5408	4	0.1352	38.6686	4.69×10^{-6}
**Within Groups (Error)**	0.0349	10	0.00349		
**Total**	0.5757	14			

Table S11: One-Way ANOVA for the effect of wt. % Hydrogen Peroxide Concentration on Foam Stability (Mean Volume Retention Ratio). $F(4, 10) = 38.6686$, $p = 4.69 \times 10-6$ ($p < 0.001$), indicating a highly significant effect of hydrogen peroxide concentration on volume retention ($n = 3$ per group).

Comparison of wt.% Hydrogen Peroxide	Difference in Means	95% Lower CI	95% Upper CI	Adjusted p-value	Significance ($\alpha = 0.05$)
1.2 − 2.4	−0.0637	−0.2230	0.0956	0.6858	No
1.2 − 3.6	−0.0556	−0.2149	0.1037	0.7768	No
1.2 − 4.8	−0.1698	−0.3289	−0.0107	0.0351	Yes
1.2 − 6.0	−0.5267	−0.6858	−0.3675	< 0.0001	Yes
2.4 − 3.6	0.0081	−0.1510	0.1673	0.9998	No
2.4 − 4.8	−0.1061	−0.2653	0.0531	0.255	No
2.4 − 6.0	−0.4630	−0.6222	−0.3039	< 0.0001	Yes
3.6 − 4.8	−0.1142	−0.2734	0.0449	0.2016	No
3.6 − 6.0	−0.4711	−0.6303	−0.3119	< 0.0001	Yes
4.8 − 6.0	−0.3569	−0.5161	−0.1977	0.0002	Yes

Table S12: Tukey's HSD Post-Hoc Test Results for Pairwise Comparisons of Mean Volume Retention Ratios (V_{24}/V_0) across different wt. % Hydrogen Peroxide concentrations. Notable: 6.0 wt. % hydrogen peroxide was significantly lower than all other concentrations; 1.2 wt. % vs 4.8 wt. % hydrogen peroxide also showed a significant difference. No significant differences among lower concentrations (1.2, 2.4, 3.6) or between (2.4, 3.6, 4.8).

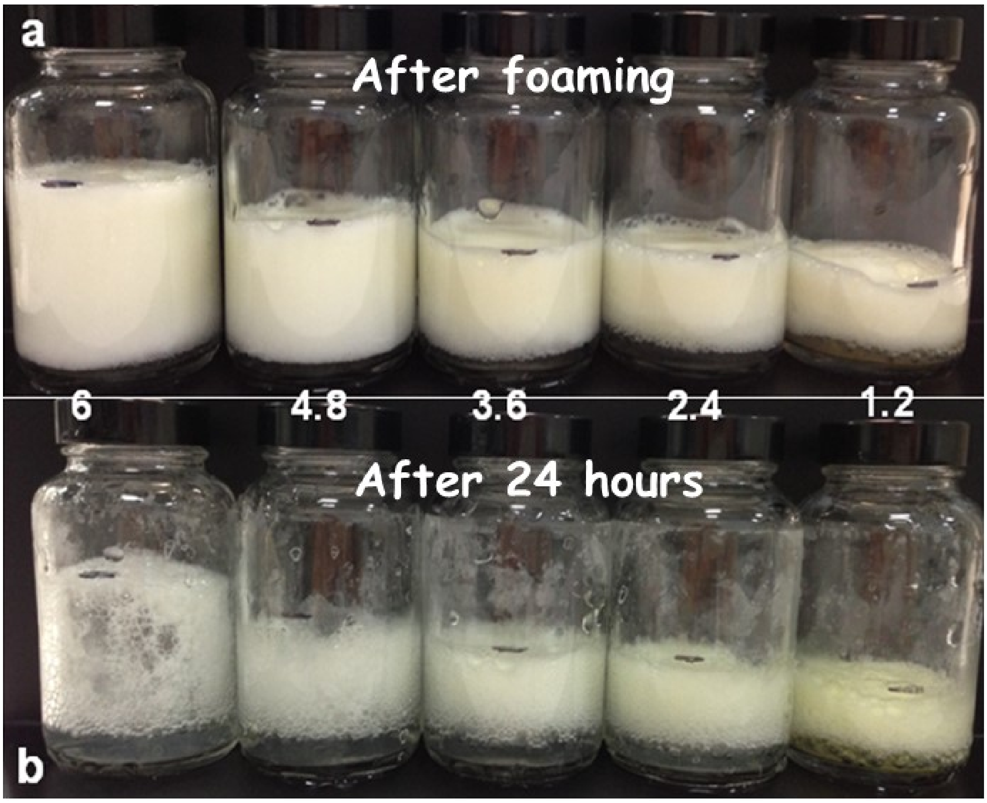

Figure S6: Visual representation of solidified foams formed at varying wt. % Hydrogen Peroxide concentrations, comparing initial state (a) and after 24 hours (b). Fixed gelatine (5.0 wt. %), catalase (0.2 wt. %), glutaraldehyde (2.5 wt. %), 37 °C.

3.1 Effect of Catalase Concentration on the Expansion Ratio of Solidified Gelatine-Based Foam:

wt. % Catalase	Vsoln = Lsoln	Vfoam-1	Vfoam-2	Vfoam-3	Expansion Ratio-1	Expansion Ratio-2	Expansion Ratio-3	Mean Expansion Ratio	SEM
0.05	0.55	2	2.2	1.8	3.64	4	3.27	3.64	0.2099
0.1	0.55	4.1	4.5	4.2	7.45	8.2	7.64	7.76	0.2251
0.2	0.55	5	5	4.9	9.1	9.1	8.9	9.03	0.0667
0.3	0.55	4.5	4.9	4.7	8.2	8.9	8.54	8.55	0.2021
0.4	0.55	4.5	4.3	4.8	8.2	7.82	8.73	8.24	0.2642

Table S13: Effect of varying catalase concentrations on foam expansion ratio. The raw data includes solution volume (Vsoln), foam volumes (Vfoam), calculated expansion ratios, mean expansion ratios, and standard error of the mean (SEM) for each catalase concentration tested. The experimental: final volume of 10 mL, gelatine concentration of 5 wt. %, hydrogen peroxide concentration of 3.6 wt. %, glutaraldehyde (GA) concentration of 2.5 wt. %, and temperature of 37 °C, with 3 replicates for each concentration.

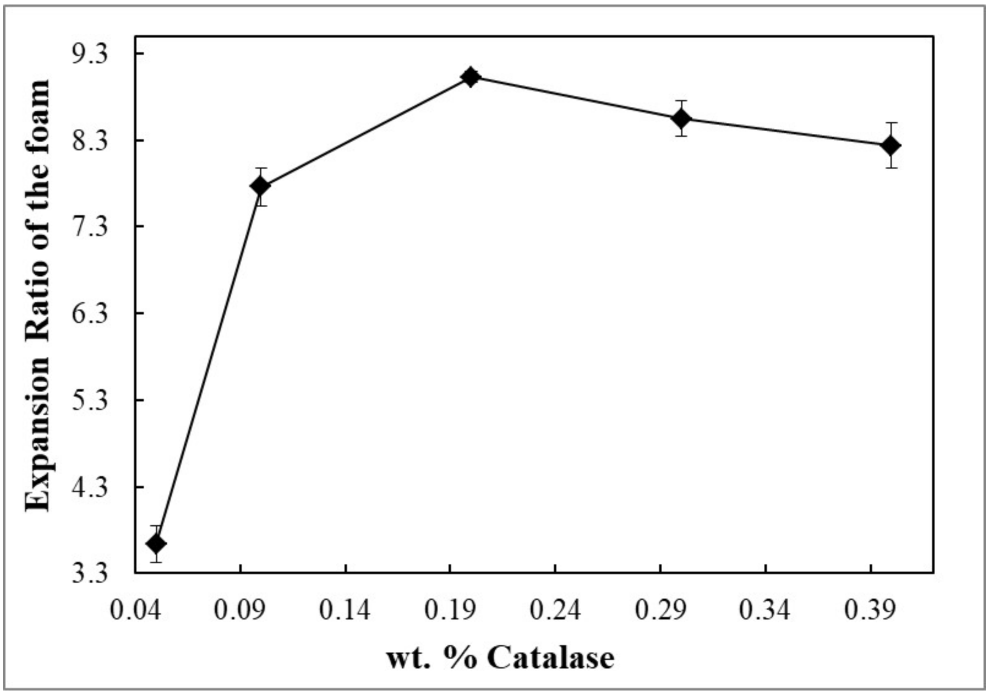

Figure S7: Effect of varying catalase concentrations on the mean expansion ratio of the foam with standard error bars as a function of catalase concentration. The experimental conditions: final volume of 10 mL, gelatine concentration of 5 wt. %, hydrogen peroxide concentration of 3.6 wt. %, glutaraldehyde (GA) concentration of 2.5 wt. %, and temperature of 37 °C, with 3 replicates for each concentration.

Source	Sum of Squares	df	F	p-value
**Catalase**	56.9677	4	113.0252	2.80×10^{-8}
**Residual**	1.2601	10	—	—

Table S14: One-Way ANOVA for the effect of wt. % Catalase Concentration on Mean Expansion Ratio. $F(4, 10) = 113.03$, $p < 2.80 \times 10^{-8}$, indicating a highly significant effect of catalase concentration on foam expansion (n = 3 per group).

Comparison of wt.% Catalase	Difference in Means	95% Lower CI	95% Upper CI	Adjusted p-value	Significance (α = 0.05)
0.05 − 0.10	−4.12	−4.87	−3.37	< 0.0001	Yes
0.05 − 0.20	−5.39	−6.14	−4.64	< 0.0001	Yes
0.05 − 0.30	−4.91	−5.66	−4.16	< 0.0001	Yes
0.05 − 0.40	−4.60	−5.35	−3.85	< 0.0001	Yes
0.10 − 0.20	−1.27	−2.02	−0.52	0.0001	Yes
0.10 − 0.30	−0.79	−1.54	−0.04	0.06	No
0.10 − 0.40	−0.48	−1.23	0.27	0.356	No
0.20 − 0.30	0.48	−0.27	1.23	0.356	No
0.20 − 0.40	0.79	0.04	1.54	0.06	No
0.30 − 0.40	0.31	−0.44	1.06	0.74	No

Table S15: Tukey's HSD Post-Hoc Test Results for Pairwise Comparisons of Mean Expansion Ratios across different wt. % Catalase concentrations. Notable: No statistically significant difference between (0.2, 0.3, and 0.4) wt. % catalase (adjusted $p > 0.05$).

3.2 Effect of Catalase Concentration on the Stability (Volume Retention Ratio V24/V0) of Solidified Gelatine-Based Foam:

wt. % Catalase	Vo-1	Vo-2	Vo-3	V24-1	V24-2	V24-3	V24-1/Vo-1	V24-2/Vo-2	V24-3/Vo-3	Mean V24/Vo	SEM (V24/Vo)
0.05	2	2.1	2.3	0.7	0.9	1	0.35	0.429	0.435	0.404	0.027
0.1	4	4.5	4.3	2	2.2	2.1	0.5	0.489	0.488	0.492	0.004
0.2	5	4.9	5.4	2.5	2.8	2.9	0.5	0.571	0.537	0.536	0.021
0.3	4.5	5.1	4.9	2.3	2.7	2.2	0.511	0.529	0.449	0.497	0.024
0.4	4.5	4.6	4.8	2.5	2.4	2.4	0.556	0.522	0.5	0.526	0.016

Table S16: Effect of Catalase Concentration on Foam Stability. Stability is expressed as the volume retention ratio (V_{24}/V_0) at fixed concentrations of gelatine (5.0 wt. %), hydrogen peroxide (3.6 wt. %), and glutaraldehyde (2.5 wt. %), in a 10 mL solution. Mean volume retention ratios are presented with standard error of the mean (SEM) (n = 3).

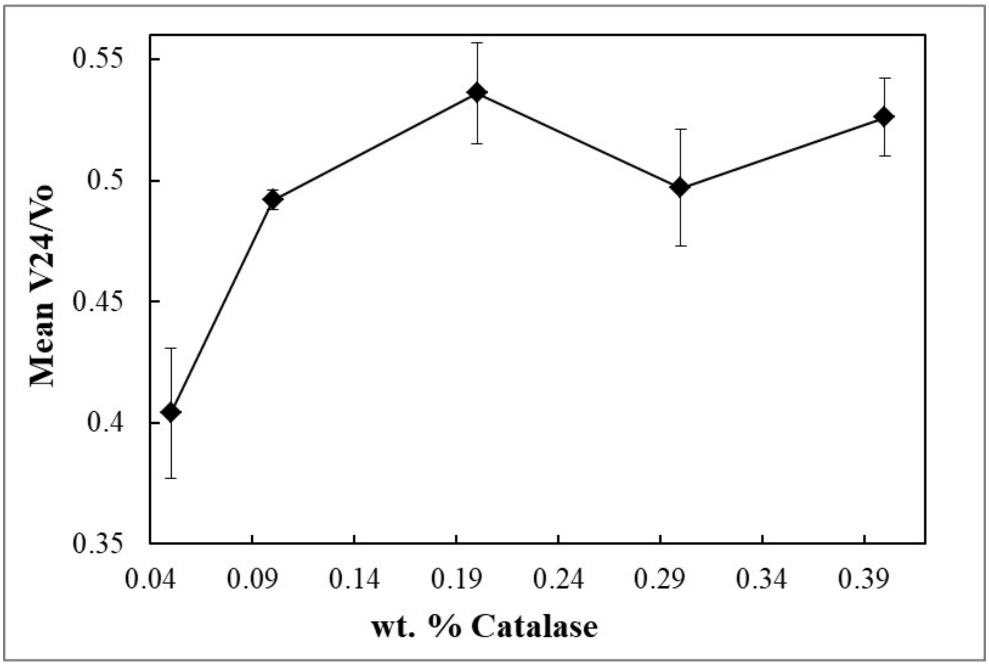

Figure S8: Effect of Catalase Concentration on the Stability of Solidified Gelatine-Based Foam. Stability is expressed as the volume retention ratio (V_{24}/V_0) at fixed concentrations of gelatine (5.0 wt. %), hydrogen peroxide (3.6 wt. %), and glutaraldehyde (2.5 wt. %), in a 10

mL solution. Mean volume retention ratios are presented with standard error of the mean (SEM) (n = 3).

Source	Sum of Squares	df	Mean Square	F	p-value
**Catalase**	0.0322	4	0.0081	6.5845	0.0073
**Residual**	0.0122	10	0.0012	—	—

Table S17: One-Way ANOVA for the effect of wt. % Catalase Concentration on Stability (Volume Retention Ratio V24/V0). $F(4, 10) = 6.5845$, $p = 0.0073$, indicating a significant effect of catalase concentration on foam stability (n = 3 per group).

Comparison of wt.% Catalase	Difference in Means	95 % Lower CI	95 % Upper CI	Adjusted p-value	Significance (α = 0.05)
0.05 vs. 0.10	0.0877	-0.0063	0.1816	0.0703	No
0.05 vs. 0.20	0.1313	0.0374	0.2253	0.0068	Yes
0.05 vs. 0.30	0.0917	-0.0023	0.1856	0.0566	No
0.05 vs. 0.40	0.1213	0.0274	0.2153	0.0114	Yes
0.10 vs. 0.20	0.0437	-0.0503	0.1376	0.568	No
0.10 vs. 0.30	0.004	-0.09	0.098	0.9999	No
0.10 vs. 0.40	0.0337	-0.0603	0.1276	0.7627	No
0.20 vs. 0.30	-0.0397	-0.1336	0.0543	0.6471	No
0.20 vs. 0.40	-0.01	-0.104	0.084	0.9962	No
0.30 vs. 0.40	0.0297	-0.0643	0.1236	0.832	No

Table S18: Tukey's HSD Post-Hoc Test Results for Pairwise Comparisons of Stability (Volume Retention Ratio V24/V0) across different wt. % Catalase concentrations. Notably, there is a statistically significant difference between 0.05 wt. % catalase and both 0.20 wt. % ($p = 0.0068$) and 0.40 wt. % ($p = 0.0114$) concentrations. No other pairwise comparisons were statistically significant (adjusted $p > 0.05$).

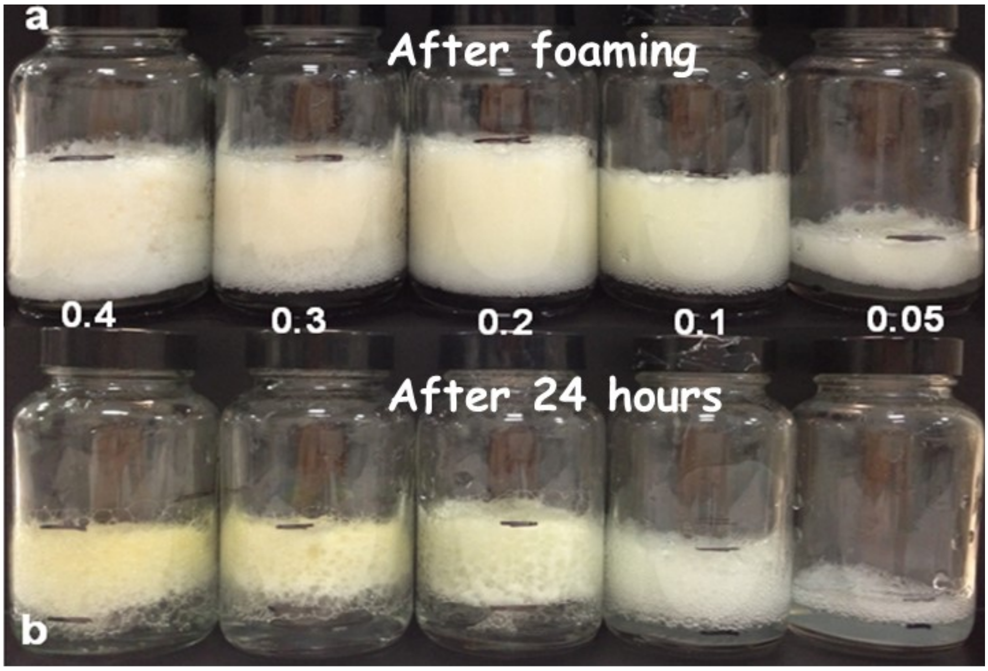

Figure S9: Visual representation of solidified foams formed at varying wt. % Catalase concentrations, comparing initial state (a) and after 24 hours (b). Fixed gelatine (5.0 wt. %), hydrogen peroxide (3.6 wt. %), glutaraldehyde (2.5 wt. %), 37 °C.

4.1 Effect of Glutaraldehyde Concentration on the Expansion Ratio of Solidified Gelatine-Based Foam:

wt. % GA	Vsoln (L)	Vfoam-1 (L)	Vfoam-2 (L)	Vfoam-3 (L)	ER-1	ER-2	ER-3	Mean ER	SEM
1	0.55	4.5	4.3	4.8	8.18	7.82	8.73	8.24	0.27
2	0.55	4	4.2	3.9	7.27	7.64	7.09	7.33	0.16
3	0.55	3.6	3.8	3.7	6.55	6.91	6.73	6.73	0.1
4	0.55	2	2.4	2.1	3.64	4.36	3.82	3.94	0.22
5	0.55	2.2	2.2	2.4	4	4	4.36	4.12	0.12

Table S19: Effect of varying glutaraldehyde (GA) concentrations on foam expansion ratio. The raw data for solution volume (V_{soln}), foam volumes (V_{foam}), calculated expansion ratios, mean expansion ratios, and standard error of the mean (SEM) for each glutaraldehyde concentration tested. The experimental conditions were as follows: final volume of 10 mL, gelatine concentration of 5 wt. %, hydrogen peroxide concentration of 3.6 wt. %, catalase concentration of 0.2 wt. %, and temperature of 37 °C, with 3 replicates for each concentration.

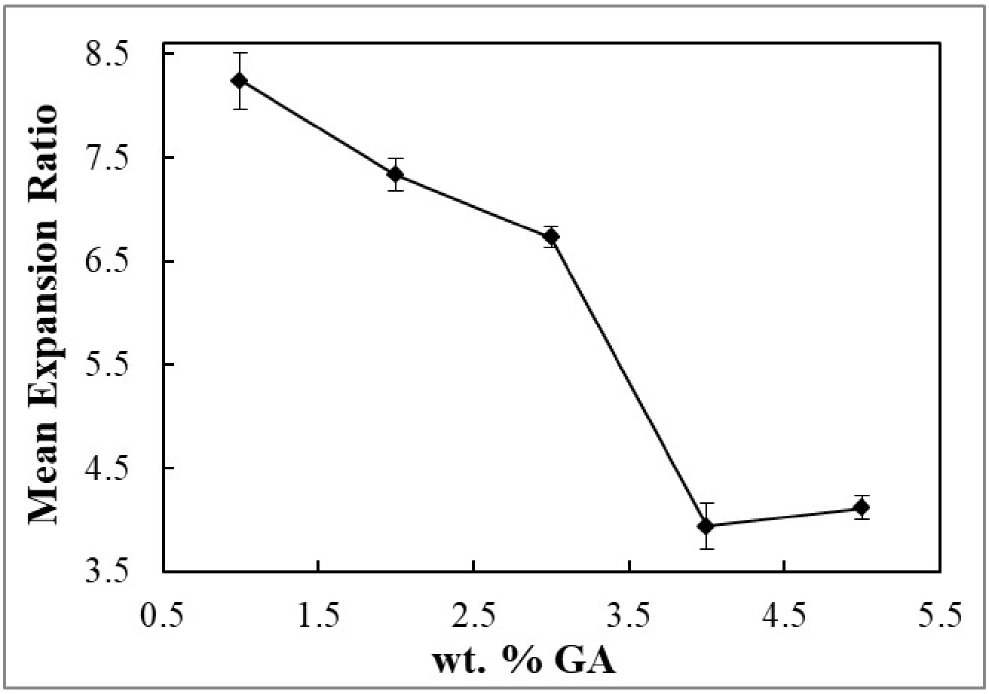

Figure S10: Effect of varying glutaraldehyde (GA) concentrations on the mean expansion ratio of the foam with standard error bars as a function of glutaraldehyde concentration. The experimental conditions: final volume of 10 mL, gelatine concentration of 5 wt. %, hydrogen peroxide concentration of 3.6 wt. %, catalase concentration of 0.2 wt. %, and temperature of 37 °C, with 3 replicates for each concentration.

Source of Variation	Sum of Squares (SS)	Degrees of Freedom (df)	Mean Square (MS)	F-statistic	p-value
Between Groups	13.69	4	3.42	112.16	2.9 x 10−8
Within Groups	0.31	10	0.03		
Total	14	14			

Table S20: One-way ANOVA for the effect of wt. % Glutaraldehyde Concentration on Mean Expansion Ratio. $F(4, 10) = 111.6$, $p = 2.97 \times 10^{-8}$, indicating a highly significant effect of glutaraldehyde concentration on foam expansion ($n = 3$ per group).

Comparison of wt.% GA	Difference in Means	95% Lower CI	95% Upper CI	Adjusted p-value	Significance (α = 0.05)
1 − 2	−0.91	−1.764	−0.056	0.0357	Yes
1 − 3	−1.51	−2.367	−0.660	0.0012	Yes
1 − 4	−4.30	−5.157	−3.450	< 0.0001	Yes
1 − 5	−4.12	−4.977	−3.270	< 0.0001	Yes
2 − 3	−0.60	−1.457	0.25	0.2137	No
2 − 4	−3.39	−4.247	−2.540	< 0.0001	Yes
2 − 5	−3.21	−4.067	−2.360	< 0.0001	Yes
3 − 4	−2.79	−3.644	−1.936	< 0.0001	Yes
3 − 5	−2.61	−3.464	−1.756	< 0.0001	Yes
4 − 5	0.18	−0.674	1.034	0.9533	No

Table S21: Tukey's HSD Post-Hoc Test Results for Pairwise Comparisons of Mean Expansion Ratios across different wt. % Glutaraldehyde concentrations. Notable: No statistically significant difference between 2.0 and 3.0 wt. %, and 4.0 and 5.0 wt. % glutaraldehyde (adjusted $p > 0.05$).

4.2 Effect of Glutaraldehyde Concentration on the Stability (Volume Retention Ratio V24 /V0) of Solidified Gelatine-Based Foam:

wt. % GA	Vo-1	Vo-2	Vo-3	V24-1	V24-2	V24-3	V24-1/Vo-1	V24-2/Vo-2	V24-3/Vo-3	Mean V24/Vo	SEM (V24/Vo)
1	4.5	4.3	4.6	2	2.1	1.9	0.44	0.49	0.41	0.45	0.02
2	4	4.2	3.8	1.8	2.2	2	0.45	0.52	0.53	0.5	0.03
3	3.6	3.8	3.5	2.2	2.1	1.8	0.61	0.55	0.51	0.56	0.03
4	2.2	2.3	2.1	1.2	1.1	1.2	0.55	0.48	0.57	0.53	0.03
5	2.2	2.2	2	1.2	0.9	1.1	0.55	0.41	0.55	0.5	0.05

Table S22: Effect of varying glutaraldehyde (GA) concentrations on foam stability ratio. The raw data for foam stability V_{24}/V_0, mean stability ratio, and standard error of the mean (SEM) for each glutaraldehyde concentration tested. The experimental conditions were as follows: final volume of 10 mL, gelatine concentration of 5.0 wt. %, hydrogen peroxide concentration of 3.6 wt. %, catalase concentration of 0.2 wt. %, and temperature of 37 °C, with 3 replicates for each concentration.

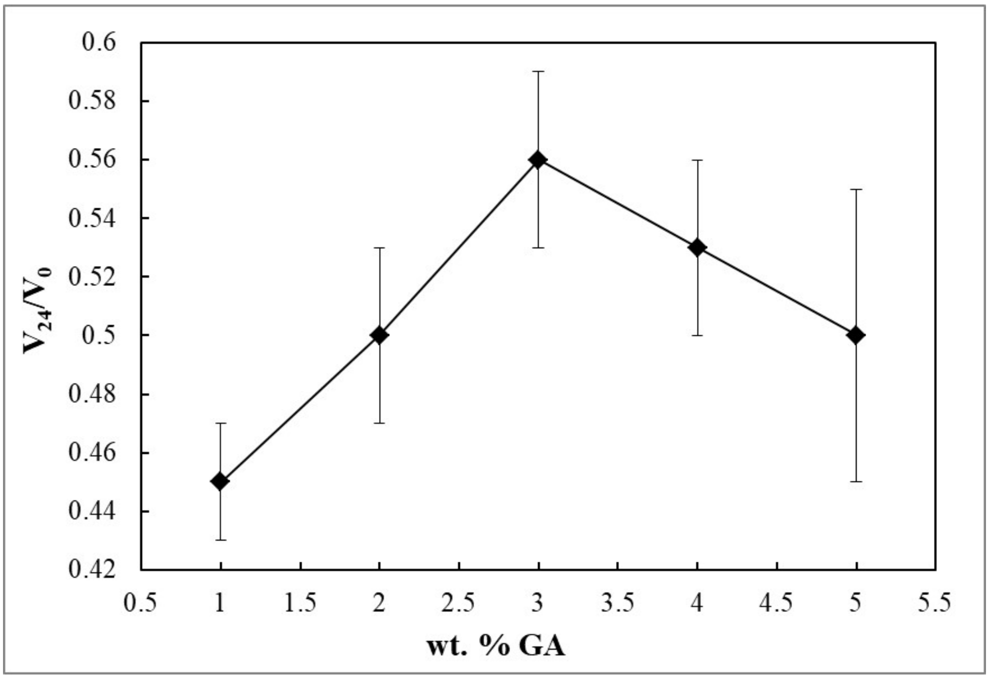

Figure S11: Effect of varying glutaraldehyde (GA) concentrations on the mean foam stability ratio (V_{24}/V_0). Error bars denote the standard error of the mean (SEM). The experiment was conducted at a fixed concentration of 5.0 wt. % gelatine, 3.6 wt. % hydrogen peroxide, and 0.2 wt. % catalase at 37 °C, with three replicates for each concentration.

Source of variation	SS	df	MS	F	p-value
Between groups	0.02057	4	0.00514	1.73	0.219
Within groups	0.02967	10	0.00297		
Total	0.05024	14			

Table S23: One-way ANOVA for the effect of wt. % Glutaraldehyde Concentration on Mean Foam Stability Ratio (V_{24}/V_0). $F(4, 10)= 1.73$, $p = 0.219$, indicating the effect of glutaraldehyde concentration on foam stability was not statistically significant at the $\alpha = 0.05$ level ($n = 3$ per group).

Comparison of wt.% GA	Difference in Means	95% Lower CI	95% Upper CI	Adjusted p-value	Significance
1 − 2	0.0533	−0.0930	0.1997	0.7521	No
1 − 3	0.11	−0.0364	0.2564	0.173	No
1 − 4	0.0867	−0.0597	0.233	0.3539	No
1 − 5	0.0567	−0.0897	0.203	0.7116	No
2 − 3	0.0567	−0.0897	0.203	0.7116	No
2 − 4	0.0333	−0.1130	0.1797	0.9393	No
2 − 5	0.0033	−0.1430	0.1497	1	No
3 − 4	−0.0233	−0.1697	0.123	0.9827	No
3 − 5	−0.0533	−0.1997	0.093	0.7521	No
4 − 5	−0.0300	−0.1764	0.1164	0.9576	No

Table S24: Tukey's HSD Post-Hoc Test Results for Pairwise Comparisons of Mean Foam Stability Ratios (V_{24}/V_0) across different wt. % Glutaraldehyde concentrations. The stability difference between 3.0 wt. % GA (highest mean stability) and 1.0 wt. % GA (lowest mean stability) had the smallest adjusted p-value (0.173), but no comparisons were found to be statistically significant at the $\alpha = 0.05$ level (all adjusted $p > 0.05$).

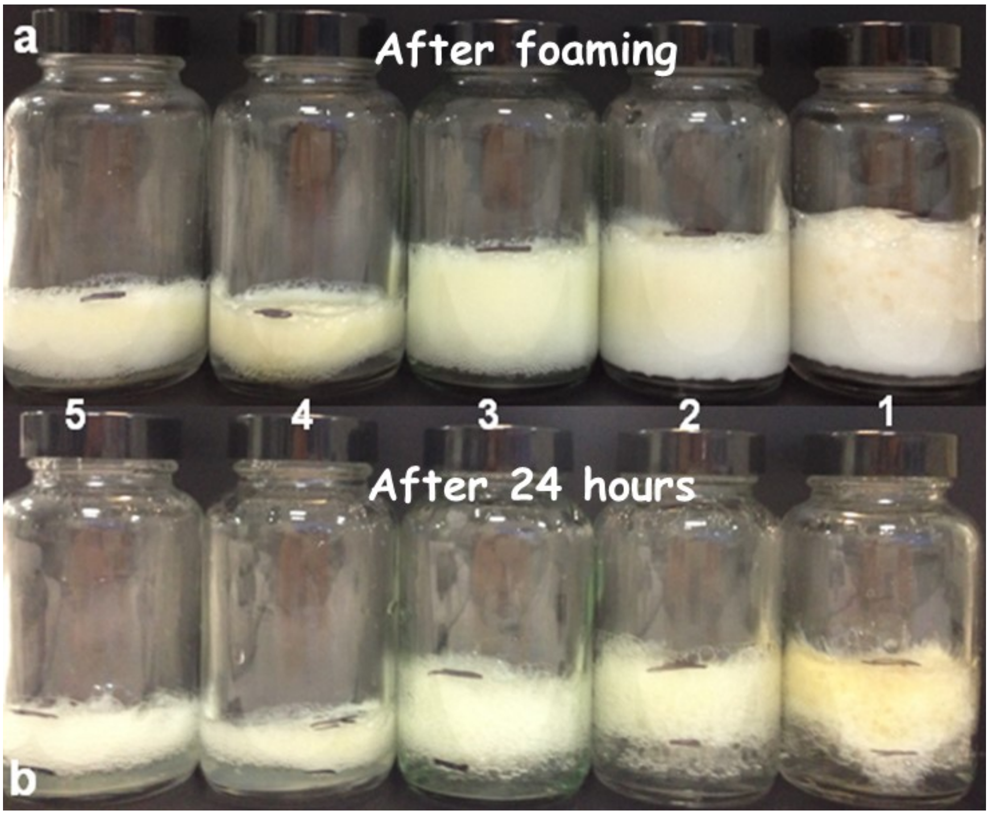

Figure S12: Visual representation of solidified foams formed at varying wt. % Glutaraldehyde concentrations, comparing the initial state (a) and after 24 hours (b). The experimental conditions were as follows: fixed gelatine (5.0 wt. %), hydrogen peroxide (3.6 wt. %), catalase (0.2 wt. %), and a temperature of 37 °C.
